# Peptide-based materials for cancer immunotherapy

**DOI:** 10.7150/thno.37194

**Published:** 2019-10-15

**Authors:** Lu Zhang, Yanyu Huang, Aaron Raymond Lindstrom, Tzu-Yin Lin, Kit S Lam, Yuanpei Li

**Affiliations:** Department of Biochemistry and Molecular Medicine, UC Davis NCI-designated Comprehensive Cancer Center, University of California Davis, Sacramento, California 95817, United States

**Keywords:** Peptide-based materials, cancer immunotherapy

## Abstract

Peptide-based materials hold great promise as immunotherapeutic agents for the treatment of many malignant cancers. Extensive studies have focused on the development of peptide-based cancer vaccines and delivery systems by mimicking the functional domains of proteins with highly specific immuno-regulatory functions or tumor cells fate controls. However, a systemic understanding of the interactions between the different peptides and immune systems remains unknown. This review describes the role of peptides in regulating the functions of the innate and adaptive immune systems and provides a comprehensive focus on the design, categories, and applications of peptide-based cancer vaccines. By elucidating the impacts of peptide length and formulations on their immunogenicity, peptide-based immunomodulating agents can be better utilized and dramatic breakthroughs may also be realized. Moreover, some critical challenges for translating peptides into large-scale synthesis, safe delivery, and efficient cancer immunotherapy are posed to improve the next-generation peptide-based immunotherapy.

## 1. Introduction

Immunotherapy is one of the most potent types of cancer therapy. It targets and eliminates cancer cells by utilizing the body's immune system and generally results in an enduring anti-tumor response and the effective regression of the cancer, while also preventing metastasis and recurrence 1-3. To achieve satisfactory immunotherapeutic outcomes, the human body requires a complex immune system that can successfully recognize and eliminate hidden cancer niches. The body's two major immune systems consist of the innate and the adaptive immune systems, with the key distinction between them being antigen specificity. Innate immunity is a nonspecific defense mechanism that reacts immediately to the appearance of antigens. Comparatively, adaptive immunity is more complicated due to antigen-specific immune responses that require the processing and recognition of an antigen. Once an antigen is recognized, the adaptive immune system induces immune cells to attack that antigen and this effect is sustained over a longer duration because of long-lived, highly specific memory T cells.

**Scheme 1:**
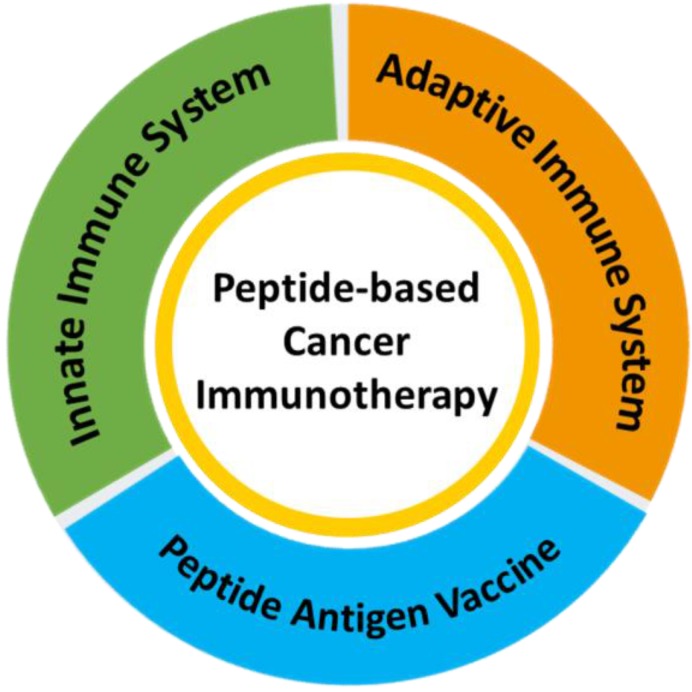
A schematic illustration of the application of peptide-based materials in diverse immune systems for cancer immunotherapy.

In recent years, peptide-based materials have been applied to solving many therapeutic problems and have shown particular efficacy as cancer immunotherapies. Peptide-based materials are unique and promising tools for cancer therapeutics and they have a variety of activities, including sensing, drug delivery, cell targeting, fate control, deep tumor tissue penetration, and the generation of immune responses for improved anti-tumor therapeutic outcomes 4-9. For example, vaccines comprised of fabricated peptides that mimic cytotoxic T-lymphocyte (CTL) epitopes are a facile and cost-efficient approach for cancer therapy. Clinical studies with peptides have indicated that they are efficient, specific, and safe for delivering therapeutic cargos into cells, while also being easy to synthesize and modify. Many of the peptides used in cancer immunotherapy have been derived from the functional domains of proteins and exert specific activities like receptor binding, stimuli responsiveness, cell penetration, and regulation of cell signaling pathways 10-14. Recently, peptides have been designed as versatile cancer vaccines that activate innate and adaptive immune systems by interacting with neutrophils, dendritic cells (DCs), macrophages, natural killer (NK) cells, T cells, B cells, *etc* 15-17. Peptides can also be used as the building blocks of advanced composites through the incorporation of specific functions like cellular targeting, responsive cleavage sites, endocytosis transporters, and therapeutic activities 18-22. Most peptide materials used for cancer immunotherapy, including immune vaccines, are designed to target specific cells within one of the two immune systems. In this review, we will provide an overview of the versatility and diversity of peptide-based materials with distinct bioactive properties and discuss their applications in cancer immunotherapy (Scheme 1).

## 2. The innate immune system

The innate immune system is composed of nonspecific defenses that can immediately resist an attack by a foreign agent upon the body. The innate immune system is comprised of a diverse variety of cells (mainly macrophages, DC, neutrophils and NK cells, etc.), which are pivotal in an immune response to a tumor.

### 2.1 Macrophages

Tumor-associated macrophages (TAM) are a normally efficient phagocytic cell type that can hunt cancer cells with fewer limits due to their ability to roam beyond the circulatory system. However, TAMs can also promote cancer proliferation and metastasis via the release of various cytokines like chemokines, inflammatory factors, and growth factors in the tumor microenvironment 23-28. Therefore, TAMs exhibit two phenotypic activation states, the antitumoral M1 and the protumoral M2. M2-TAMs are immunosuppressed and promote tumor invasion, which has made them the primary target of macrophage-focused cancer immunotherapies. Peptide-based cancer immunotherapies have primarily aimed to block M2-TAM activities by impeding the recruitment of macrophages to tumors and switching M2-TAMs into M1-TAMs. More impressively, multiple peptide-based materials that target TAMs have progressed into early clinical trials 29-31.

**Figure 1:**
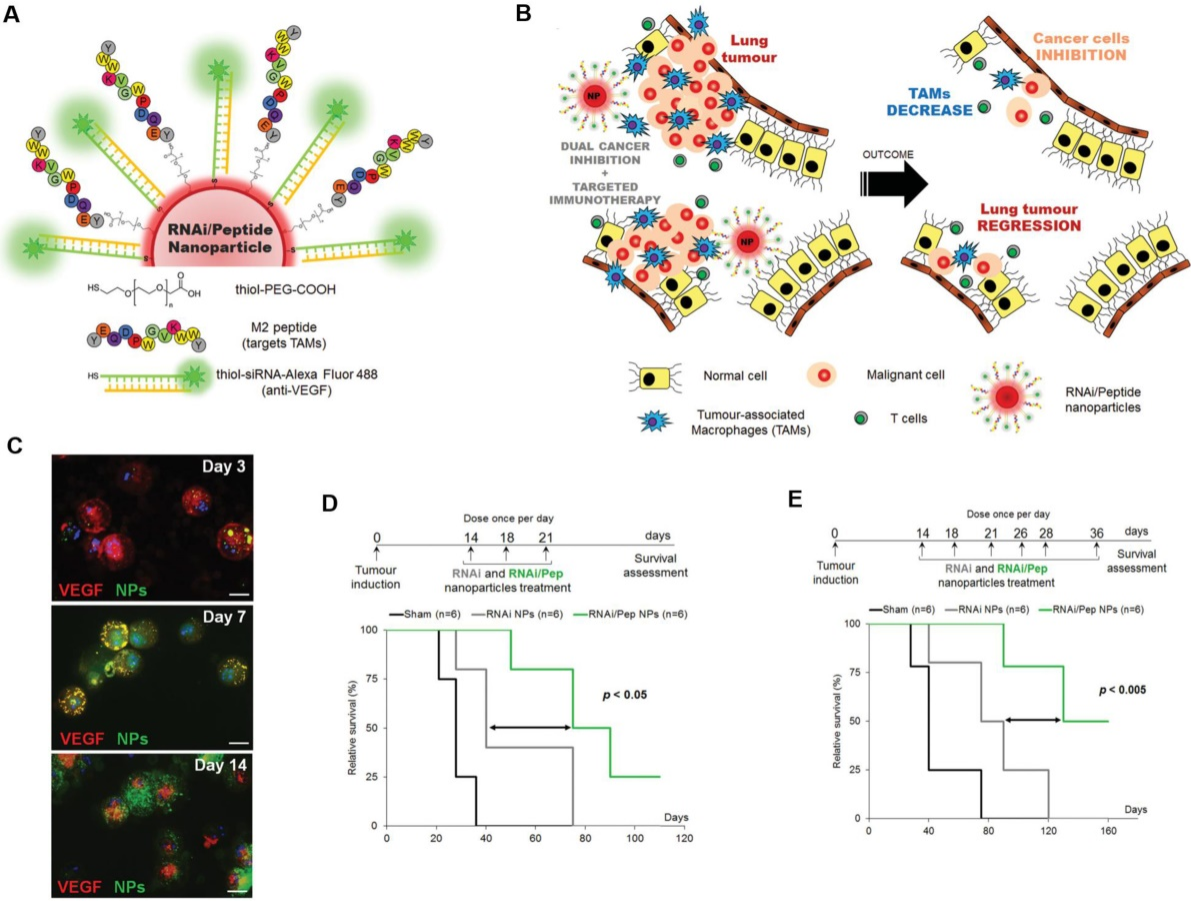
TAM-targeted immunotherapies for the treatment of cancer. **A,B)** Schematic of the combined siRNA-silencing therapy and immunotherapy targeting TAMs and cancer cells *in vivo* using RNAi/Peptide nanoparticles (NPs) administered directly to bronchial airways. **C)** Immunofluorescence microscopy images of recovered macrophages from BAL fluid show RNAi/Peptide NPs (green) internalization and VEGF (red) expression. **D,E)** Survival curves of untreated control (sham, in black) and RNAi NPs (gray) and RNAi/Peptide NPs (green) mice using two dosages. Adapted with permission from 32, copyright 2015, Wiley-VCH.

**Figure 2:**
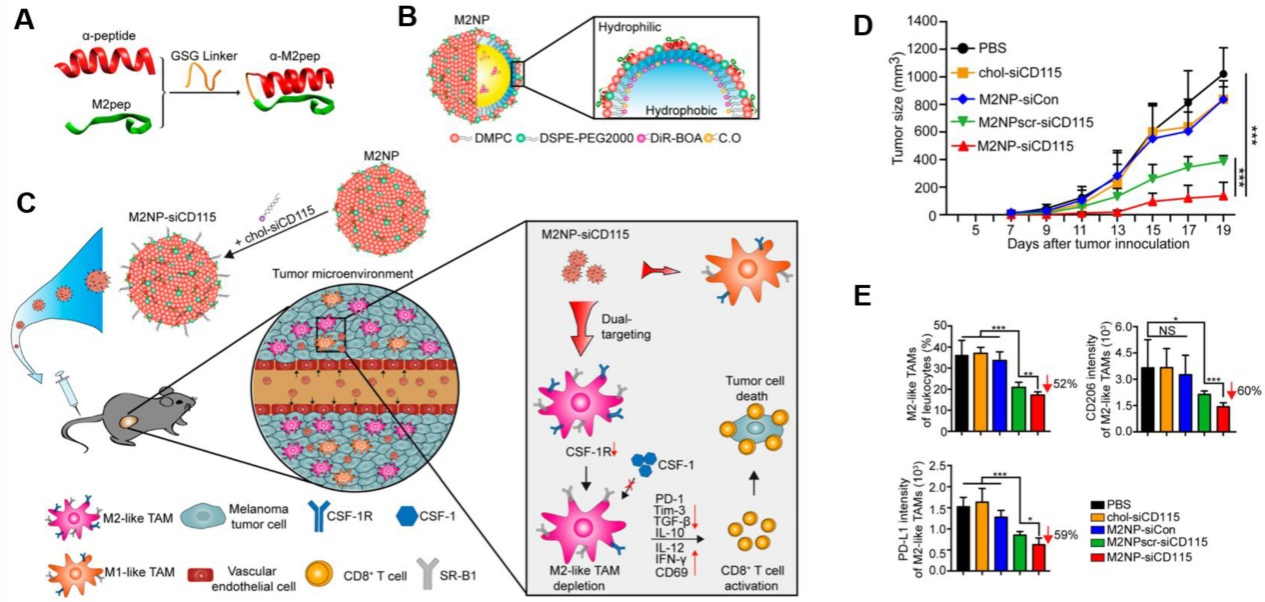
M2NP-based M2 TAM-specific molecular-targeted immunotherapy. **A)** Hybrid approach of the fusion peptide α-M2pep. **B)** Structure and components of M2NP. **C)** M2NP-based delivery of siRNA for CSF-1R silencing and immune regulation *via* synergistic dual targeting of M2-TAMs *in vivo*. **D)** Tumor growth curves of B16 tumors in C57BL/6 mice treated with different groups. **E)** Proportion of M2-TAMs among the total tumor infiltrating leukocytes in mice, the CD206 expression by M2 TAMs and the PD-L1 expression on M2-TAMs after the indicated treatment. Adapted with permission from 33, copyright 2017 American Chemical Society.

Targeting TAMs with an M2-specific peptide has shown promising antitumor activity by selectively delivering potent therapeutics directly to M2-TAMs. João Conde *et al.* developed a biohybrid vascular endothelial growth factor (VEGF) mRNA interference-M2 targeting peptide (YEQDPWGVKWWY) material with the ability to immune-modulate TAM cell populations. This peptide-based immunotherapy blocked both M2-TAM activity and cancer cell growth by inhibiting VEGF-associated signaling pathways and triggering a host immune response that caused sustained tumor regression. Their formulation caused specific and enduring therapeutic effects in inflammatory TAMs and the production of anti-tumor immune memory dramatically inhibited tumor progression for extended periods (Figure 1) 32. Yuan Qian *et al*. developed a dual-molecular-targeted immunotherapeutic consisting of a M2-TAM peptide (M2pep: YEQDPWGVKWWY) and an α-peptide (a scavenger receptor B type 1 (SR-B1)) that they named M2NP. This combination specifically blocked the survival signal of M2-TAMs in melanoma tumors and led to a significant depletion of M2-TAMs within the tumor. It also inhibited IL-10 and transforming growth factor-β (TGF-β) expression, while escalating IL-12 and IFN-γ expression in the tumor microenvironment (Figure 2) 33. Highly compelling TAM depletion peptides were developed by Chayanon Ngambenjawong *et al*. by boosting the valency of the targeting M2pep and KLA (pro-apoptotic peptide) drug domains. Both [M2pep]_4_-Biotin and [M2pep]_2_-[KLA]_2_ exhibited specific toxicities towards M2-TAMs and cancerous cells, while sparing M1-TAMs 34. Kuan-Yin Shen *et al.* reported a mono-palmitoylated peptide that could induce an anti-cancer immune response without the addition of an adjuvant. Later, they developed a di-palmitic acid-conjugated long peptide with Toll-like receptor 2 (TLR2) agonist properties that improved anti-tumor immunity by diminishing the function of TAMs 35. Additionally, Meiyu Peng *et al*. developed an apolipoprotein mimetic peptide that reduced the proportion of M2-TAMs within a tumor by decreasing the expression of the M2 marker genes, such as Arg1, IL-6, iNOS, and IL-12 36. The addition of a cell penetrating peptide (RKKRRQRRR) can enhance the delivery of siRNA immunotherapies targeting TAMs. Wei Bin Fang *et al*. developed a novel gene silencing Ca-TAT cell penetrating peptide/CCL2 siRNA complex that not only decreased the recruitment of M2-TAMs but also reduced stem cell renewal and inhibited of tumor growth and metastasis 37.

Using peptides as a “protection strategy” in nanomaterial design can avoid unwanted macrophage-mediated clearance of the nanomaterial before it reaches the target. The membrane protein CD47, a "marker of self" in humans and mice, interacts with the phagocyte receptor CD172a on macrophages to inhibit the phagocytosis of cells. Pia L. Rodriguez *et al.* designed minimal "Self" peptides based on human CD47 and attached them to particles, which effectively inhibited the macrophage-mediated clearance of nanoparticles from the body 38. Recently, using T-cell independent targeting of macrophages *via* PD1-inhibition was reported to trigger enhanced macrophage function in the adaptive immune response 3, 39. These results suggest that a peptide-based TAM-targeting approach is a promising adjunct strategy to add to the arsenal of cancer immunotherapies.

### 2.2 Dendritic cells (DCs)

DCs were originally identified by Steinman *et al*. in 1972 and are one of most dominant types of antigen-presenting cells (APCs). These cells process antigens and present them on their cellular surface for recognition by T cells. Once cancer cells are identified by the immune system, DCs residing in all peripheral tissues can recognize antigens and raise alerts. DCs play an essential role in activating CD4^+^ and CD8^+^ T cells that will induce specific cytotoxic responses in cancer cells. DCs can express CD40, CD80, CD86, major histocompatibility complex (MHC), and toll-like receptors (TLR) that are important in the presentation of peptides to T and B lymphocytes. DCs also possess the unique capacity to initiate primary immune responses, which has resulted in the DC-based peptide delivery of tumor antigens that have shown promising results as cancer immunotherapies.

Peptides loaded into DCs can induce anti-tumor immunity and form a foundation for the optimization of peptide-based immunotherapies against cancer 40. DC-mediated peptide delivery has been demonstrated on multiple occasions to have clear immunomodulating effects 41. One target for peptide delivery to DCs is Clec9a, which is specifically expressed on mouse CD8^+^ and CD103^+^ DC subsets and is responsible for antigen cross-presentation. Using a phage display technique, Zhongyi Yan *et al*. identified a peptide that was capable of specifically binding to mouse Flt3L-induced Clec9a^+^ DCs and Clec9a over-expressing HEK-293T cells. When coupled with an ovarian epitope, this fusion peptide greatly improved the ability of Clec9a^+^ DCs to stimulate CD8^+^ T cells and decreased lung metastasis 42. Rong-Fu Wang *et al*. designed a strategy to prolong the presentation of an MHC class I-restricted self-peptide on DCs by linking it to a cell-penetrating peptide (CPP). Their peptides were derived from tyrosinase-related protein 2 and covalently linked to a CPP sequence (AAVLLPVLLAAP). These peptides were loaded in DCs and were able to activate T cells for over 24 h. Treatment with these DC-loaded peptides completely protected immunized mice from tumor progression and drastically suppressed lung metastases 43. Yoo Jin Choi *et al*. utilized peptides from cancer stem-like cells as antigens to trigger DC vaccination against both human breast cancer and hepatocellular carcinoma. By pulsing DCs with CD44 and EpCAM based peptides, they were able to effectively stimulate the production of mature DCs, enhance T cell stimulation, and increase the number of CTLs 44.

Naked peptides are widely used in DC vaccines as targeted peptide antigens and can be loaded into an amplified DC *in vitro* before its reintroduction to the patient. By loading DCs with an tumor-associated epitope, these DC vaccines can induce the production of antigen-specific T cells with multiple specificities that can inhibit tumor growth 40. DC vaccines loaded with the HLA-A24 peptide (CEA652) could prevent further tumor growth and decrease the levels of carcinoembryonic antigen (CEA) in serum 45. The selective expression of the MAGE gene in melanomas and gastrointestinal cancer tissues has made the design of MAGE-based peptides a novel focus for the development of treatments for gastric carcinomas. Autologous DCs pulsed with a MAGE-3 peptide (four times every 3 weeks) displayed some positive results in patients with advanced gastrointestinal carcinoma. These include peptide-specific CTL responses, improvement in performance status, decreases in tumor markers, minor tumor regressions, and no evidence of toxic side effects 46. Despite the promising initial data on the use of MAGE peptides to vaccinate patients 47, Johan Vansteenkiste *et al.* reported that there was no significant difference in disease-free interval compared to the control group 48, 49. Lawrence Fong *et al*. loaded CEA-derived peptides into DCs to create a cellular vaccine that increased the CD8^+^ T cell population within tumors. There was a significantly positive clinical response for patients after vaccination with this DC vaccine, as 5 out of 12 patients showed at least inhibition of further tumor growth 50.

Despite all this promising work, DC-loaded peptide-based materials are still limited in their ability to launch anti-tumor T-cell responses, even with the aid of a potent adjuvant. Major limitations in current DC-loaded peptide therapies include the insufficient recruitment of host DCs, low DC cell viability, the transient duration of transplanted DCs at the vaccination site, and cancer-induced dysfunction of DCs. Dysfunction induced by cancer plays an especially important part in regulating the antitumoral immunotherapeutic outcomes 51.

### 2.3 Neutrophils

**Figure 3:**
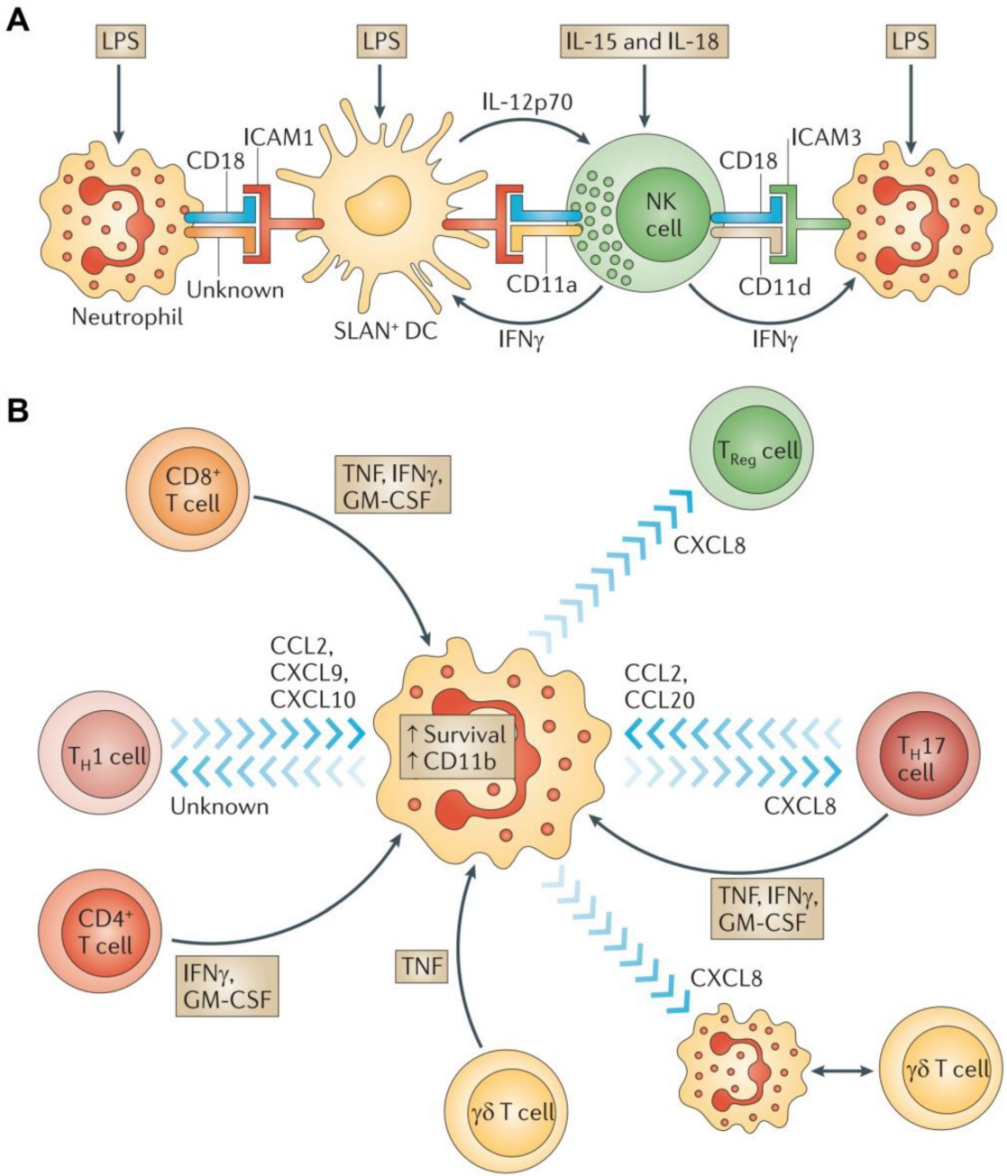
Neutrophils interactions with other immune cells. **A)** Crosstalk between neutrophils, NK cells, and SLAN+ DCs. **B)** Interplay between neutrophils and T cells. Adapted with permission from 57, copyright 2011 Springer Nature.

**Figure 4:**
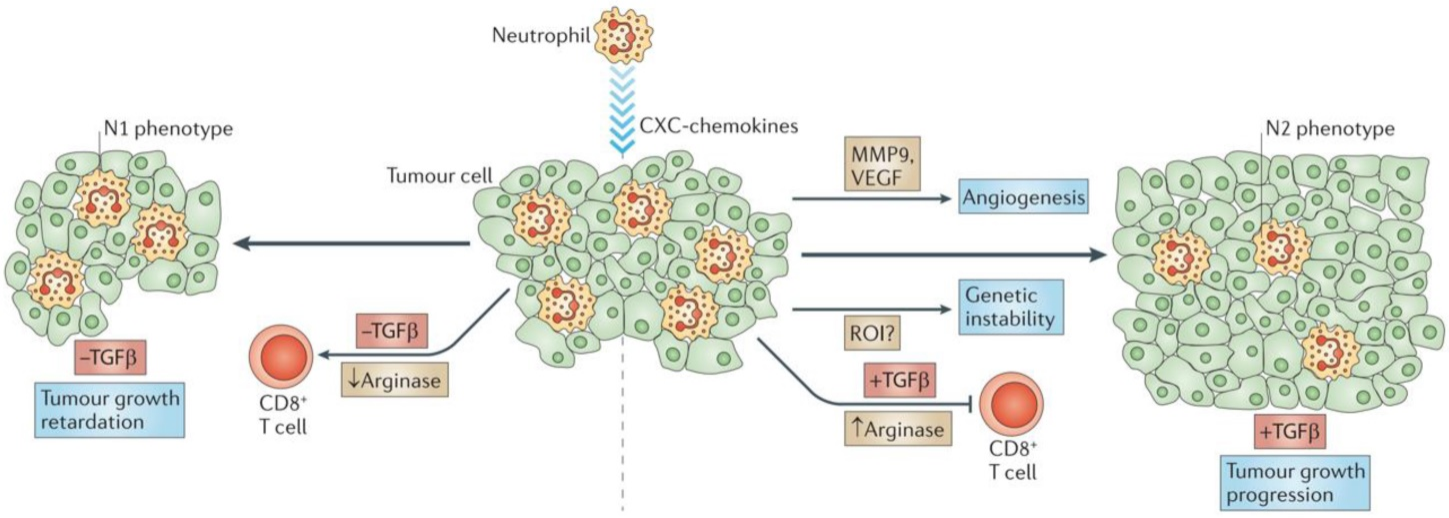
Tumor-associated neutrophils. Neutrophils are driven by TGF-β to acquire a polarized, pro-tumoral N2 phenotype. In the presence of N1 neutrophils, CD8+ T cell activation increases, and this results in more effective anti-tumor activity. Adapted with permission from 57, copyright 2011 Springer Nature.

Neutrophils are important players in innate responses for the immunotherapy of numerous cancers. When stimulated by chemokines, lipid metabolites, and danger-associated molecular patterns, neutrophils migrate to sites of inflammation and elicit inflammatory responses to defend against invading pathogens 52. Neutrophils are typically the first cells to arrive at a tumor site because of the large number of them in circulation at any given time. Once recruited into tissues, neutrophils interact with macrophages, mesenchymal stem cells, DCs, NK cells, B cells, and T cells as they engage in the eradication of cancerous cells (Figure 3). Anti-tumor neutrophils eliminate tumor cells by engulfing them and bathing them in a variety of cytotoxic substances capable of destroying the cells 53. Activated neutrophils also export proteinases into the extracellular environment that can cause damage to the surrounding host tissues 54. Besides these cytotoxic responses, neutrophils can release cytokines and chemokines to regulate inflammatory cell recruitment and immune responses to various challenges 55. These processes also lead to the recruitment of polymorphonuclear leukocytes and activate them within the tumor microenvironment (Figure 4) 56, 57.

However, the role of neutrophils in cancer development remains controversial. Several specific neutrophil responses may promote tumor progression through a number of signaling pathways and interactions with tumor, inflammatory, and stromal cells in the tumor microenvironment 52. The identification of polymorphonuclear myeloid-derived suppressor cells (PMN-MDSCs) with immunosuppressive and pro-tumorigenic activities has helped to partially resolve this controversy. Also, some neutrophils have been deemed MDSC-like due to their favoring of tumor proliferation without causing immunosuppressive activity when they accumulate within a tumor.

On the other hand, the diverse nature of the tumor microenvironment can have varied effects upon the actions of tumor-associated neutrophils (TAN). Changes in the expression of genes encoding cytokines, chemokines, adhesion molecules, and granule-associated proteins within the tumor microenvironment allow TANs to undergo an “alternative activation” 58. Interestingly, exposure of neutrophils to regulatory factors like granulocyte-colony stimulating factor (G-CSF) and TGF-β can transform them into pro-tumor (N2) phenotype, while the presence of interferon-β (IFNβ) or the suppression of TGF-β induces TANs to express an antitumor (N1) phenotype. N2 neutrophils induce immunosuppression in the tumor microenvironment and have a number of associated markers, including CC-chemokine ligand 2 (CCL2), CCL5, neutrophil elastase (NE), cathepsin G, and a higher expression of arginase. Conversely, N1 neutrophils have elevated expression of immuno-activating chemokines and cytokines like TNF, ICAM-1, and FAS.

Radical oxygen species (ROS)-associated neutrophils have demonstrated potent genotoxic and cytotoxic effects that have led to significant tumor regression. NADPH oxidases in neutrophil phagolysosomes reduce molecular oxygen into superoxide radicals (O_2_^-^) that are eventually converted by the neutrophil-specific enzyme myeloperoxidase into hypochlorous acid. Interestingly, Alyssa Gregory and A.M. Houghton produced antitumor “N1” cells by depriving them of TGF-β and these N1 cells expressed larger amounts of TNF-α, nitric oxide, H_2_O_2,_ and MIP-1α compared with N2 TANs. This change in TAN activity resulted in significant tumor cell cytotoxicity and reduced tumor proliferation 56, 59.

Strategies designed to target TANs, circulating neutrophils, and myeloid regulatory cells are considered promising paradigms for next generation immunotherapies and recent progress in peptide-based materials has enabled their ability to modulate neutrophil activity. G-N. Liu *et al*. investigated the presence of an epithelial neutrophil-activating peptide (ENA-78), myeloperoxidase, and NE in pleural effusions and their chemoattractant activity on neutrophils 60. Since NE can be rapidly taken up by tumor cells lacking endogenous NE expression, peptides derived from NE are being designed as immunotherapies in solid tumor malignancies. NE uptake by tumors is regulated by neuropilin-1, which is broadly expressed in tumors. This implies that neuropilin-1 may be a useful target for immunotherapy strategies that target cross-presented antigens 61. Luca Mazzucchelli *et al*. identified cell-binding peptide sequences that bound specifically to the membrane surface of human neutrophils or monocytes using phage display libraries 62. Heini M. Miettinen *et al*. reported that peptide-nanoparticles bound specifically to neutrophils in human and mouse blood by recognizing CD177, a neutrophil-specific surface marker 63.

Selective tumor-associated molecules that modulate neutrophil infiltration into tumors have provided specific therapeutic targets for new cancer immunotherapies. Based on the elevated concentrations of Human Neutrophil Peptides-1, -2 and -3 (HNP 1-3) in the serum of colon cancer patients, HNP 1-3 are believed to serve as crucial blood markers for the detection of colon cancer. Jakob Albrethsen *et al*. demonstrated that HNP1-3 could cause apoptosis in MDCK cells *in vitro* and proposed that HNP 1-3 were carried into the bloodstream by attaching to plasma proteins in the tumor microenvironment 64. HNP-1 is known to possess antimicrobial activities but is upregulated in many cancers and has implied roles in both the tumor microenvironment and within cancer cells. Diana Gaspar *et al*. demonstrated that HNP-1 preferentially bound to cells from solid human prostate adenocarcinomas, induced cell membrane defects, and caused apoptosis at low concentrations (Figure 4) 57, 65. Intriguingly, cancer cell membranes commonly contain the a2-isoform of vacuolar ATPase (a2V) and the secretion of the N-terminal domain of a2V (a2NTD) induces a pro-tumoral phenotype of neutrophils. Safaa A. Ibrahim *et al*. reported that the treatment of human neutrophils with recombinant a2NTD led to neutrophil adherence and polarization, which suggests that a2V is a direct modulator of neutrophil migration 66.

### 2.4 Natural Killer cells

**Figure 5:**
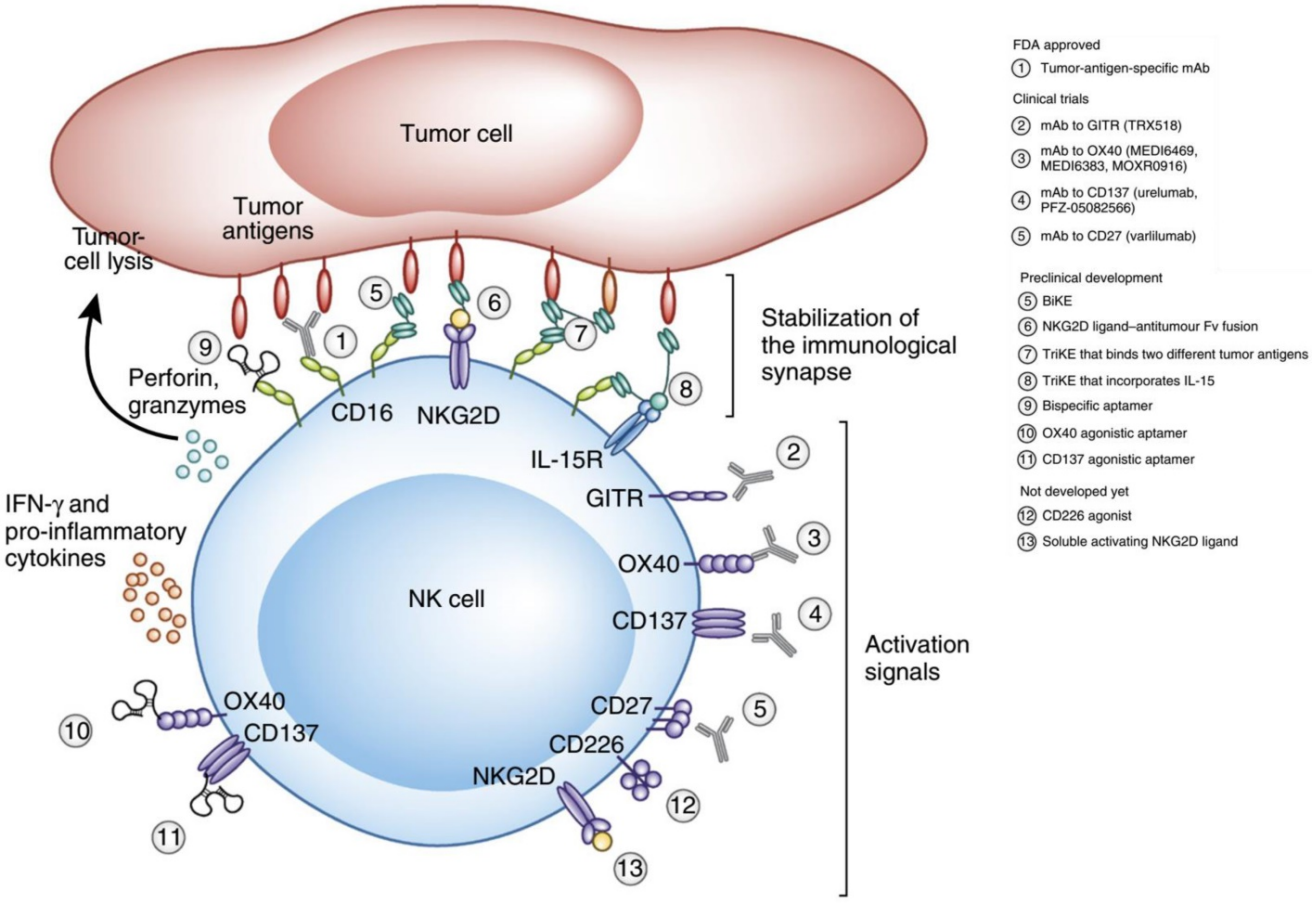
Therapeutic approaches that engage activating receptors on NK cells. Adapted with permission from 69, copyright 2016 Springer Nature.

NK cells are cytotoxic lymphocytes that are crucial to the functions of the innate immune system 67. NK cells rapidly respond to tumor formation because of their ability to recognize stressed cells without the aid of antibodies and MHC. This property of NK cells is crucial in cancer immunotherapy because many tumor cells lack MHC I markers, which prevents them from being recognized and eliminated by other immune cells. NK cell receptors fall into several categories based on their function and are classified as the natural cytotoxicity receptor, MHC-independent receptor, activating receptors (Ly49, NCR, CD16), and inhibitory receptors (Killer-cell immunoglobulin-like receptors (KIRs), CD94/NKG2). NK cell activation is controlled by a balance between inhibitory and activating stimulations. NK cell activity is upregulated when activation signaling takes precedence, while their activity is impeded when the inhibitory receptor signaling is more prominent (Figure 5) 68, 69.

KIRs are inhibitory NK cell receptors that have been demonstrated to affect the therapeutic outcomes of a number of cancer treatments. Anaïs Chapel *et al*. reported that an HLA-C*06:02-presented peptide could bind to the NK activating receptor KIR2DS1, which was sufficient for the activation of primary KIR2DS1(+) NK cells 70. A nonamer peptide (VAPWNSLSL) derived from TIMP1 was developed by Sorcha A. Cassidy *et al*. that was able to induce the binding of both KIR2DL2 and KIR2DL3 to HLA-Cw*0102 and inhibited NK cell activation 71. Since CD94 is known to form heterodimers with either NKG2A (the inhibitory receptor) or NKG2C (the activating receptor), Kuldeep S. Cheent *et al*. designed an HLA‐G leader peptide (VMAPRTLFL) to strongly inactivate CD94:NKG2A^+^ and CD94:NKG2C^+^ NK cells. A 1 μM concentration of the HLA-G leader peptide was adequate to inactivate NKG2A^+^ NK cells by the stabilization of HLA‐E on the TAP‐deficient cell line 721.174 72. Quirin Hammer et al. developed several peptides (VMAPRTLFL, VMAPRTLIL, VMAPRTLVL), which could manipulate the stimulation of NKG2C+ NK cells. These peptides were further able to regulate the generation of NKG2C+ NK cells and could induce the accumulation and differentiation of NKG2C+ NK cells from HCMV-seronegative donors 73.

Despite the activity of NK cells as effectors of innate immunity, recent studies have found that both activating and inhibitory NK cell receptors play crucial roles in self-tolerance and preservation of NK cell function. Notably, NK cells also participate in the adaptive immune response. Extensive studies have proven that NK cells can also formulate antigen-specific immunological memory and play a central role in responding to secondary infections with the same antigen 74, 75.

## 3. The adaptive immune system

The adaptive immune system, also known as the acquired immune system, is a more sophisticated and specific defense against foreign infections than the innate immune system and is imbued with potent anti-tumor attributes. The adaptive immune system is mainly composed of T cells (including CD4^+^ helper and CD8^+^ cytotoxic T cells) and B cells. CD4^+^ helper T cells release cytokines to manipulate the function of innate cells, NK cells, B cells, and CD8^+^ killer T cells to allow them to recognize and eliminate abnormal cells. Many current cancer immunotherapies, like checkpoint blockade antibodies and adoptive T cell transfer, rely on the ability of cytotoxic CD8^+^ T cells to infiltrate tumors and destroy cancer cells 76. B cells also play an important role in vaccine responses by generating antibodies that recognize specific antigens and either inactivate the antigen or mark it for destruction. In the adaptive immune system, T or B cells bind an antigen, which then promotes the reproduction of the antigen-specific cells that can search and destroy the targeted antigen. These immune cells also play a central role in supporting innate immune defenses against tumor cells. After a tumor has been cleared, most of these cells (∼90%) undergo programmed cell death, while a small fraction of differentiated memory cells are left behind to provide a rapid response if the same tumor cells appear again in the body 77. This trait is called immunologic memory and is a hallmark of the adaptive immune system 78. These immunological memory cells also express clonal antigen receptors that allow for the recognition of various antigens commonly expressed by cancer cells. When exposed to these antigens, memory cells can quickly replicate to create large numbers of effector cells in response to the new challenge to the immune system.

### 3.1 T Cells

There are three types of mature T cells: Cytotoxic T cells, Helper T cells, and T regulatory (Treg) cells. Cytotoxic CD8^+^ T cells are responsible for eliminating pathogens and tumor cells. Helper CD4^+^ T cells are responsible for activating cytotoxic T cells, B cells, and other immune cells. Treg cells secrete CD4^+^, CD25, and the transcription factor Foxp3, which help discriminate between endogenous and exogenous substances to reduce the risk of autoimmune responses.

Cytotoxic CD8^+^ T cells are responsible for killing cancer cells because they can detect and attack cancer cells that present tumor-specific antigens. However, this process can be blocked by inhibitory receptor ligands like PD-L1 or PD-L2 expressed on cancer cells. Moreover, activation of CD8^+^ cytotoxic T-cells is associated with the generation of CD4^+^ regulatory T cells that inhibit the activity of effector T cells 79, 80. Checkpoint blockade immunotherapy is one of the newest therapeutic approaches to help restore the immune system's ability to find and attack hidden cancer cells 81-87. In clinical trials, the blockade of immune checkpoints like CTLA-4 and PD-1 has proven to be a successful and efficient therapeutic method for the treatment of multiple cancers. Several strategies have been employed to precisely target tumor cells through checkpoint blockade immunotherapy. For example, anti-CTLA-4 antibodies modulate helper T cell activity by boosting effector T cell activity while downregulating Treg immunosuppressive capacity. The FDA has approved 6 antibodies that target the PD-1/PD-L1 pathway, with atezolizumab, durvalumab and avelumab targeting PD-L1, and Cemiplimab, nivolumab and pembrolizumab targeting PD-1 for the treatment of a diverse array of cancers. Although these antibodies have made great progress as cancer treatments, their application in patients remains limited due to high production costs. Peptides with similar specific binding properties to these antibodies have the advantage of much lower production costs and amenability to chemical synthesis. Chunlin Li *et al*. identified a PD-L1 targeted peptide (SGQYASYHCWCWRDPGRSGGSK) with high affinity through bacterial surface display methods and the ability of the peptide to inhibit the interaction of PD-1 with PD-L1 was verified *in vitro*. The peptide was able to retard tumor growth in mice to a larger degree than a PD-L1 antibody (56% vs. 71% respectively), suggesting that this peptide is at least as efficacious as antibody-based therapies 88. Another antagonist of the PD‐1/PD‐L1 signaling pathway was identified by Hao‐Nan Chang *et al*. by the use of a mirror‐image phage display. Their hydrolysis-resistant D-peptide (FPNWSLRPMNQM) antagonist was able to inhibit the PD-1/PD-L1 interaction both *in vitro* and *in vivo* 89.

Though immune checkpoint inhibitors can bolster the immune system's response to cancer cells, the associated adverse reactions to their use can cause severe and irreversible damage to multiple organs. Common side effects of immune checkpoint inhibitors include rash, diarrhea, low thyroid hormone, and fatigue, which will cause inflammation of the lung, intestines, liver, kidney, heart, or neurological system.

**Figure 6:**
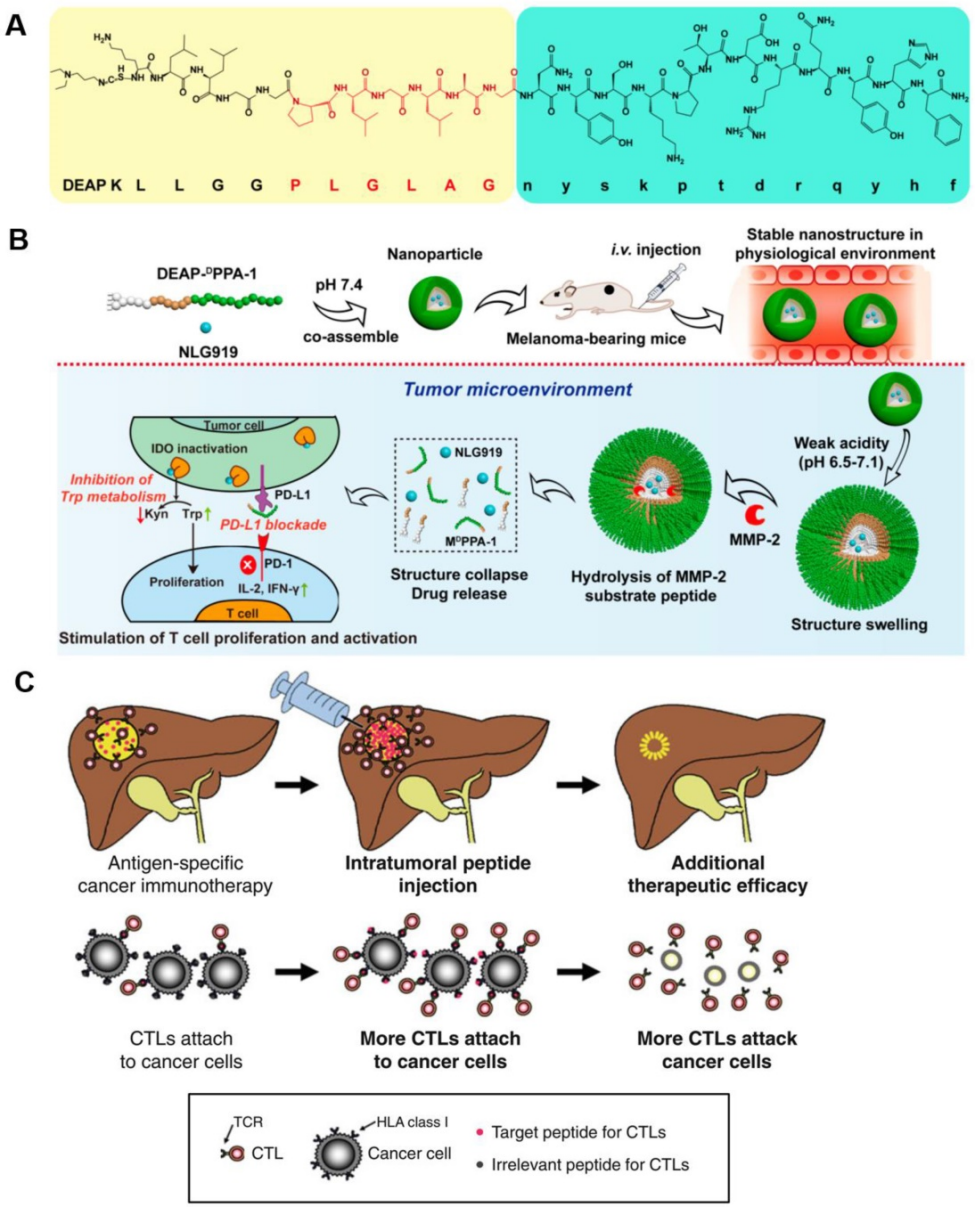
The inhibitory effects of DEAP-DPPA-1 nanoparticles on melanoma growth and their ability to induce an antitumor immune response. **A)** Composition of DEAP-DPPA-1. **B)** The antitumor mechanism of NLG919@DEAP-DPPA-1 nanoparticles. Adapted with permission from 90, copyright 2018 American Chemical Society. **C)** A mechanistic model of intratumoral peptide injection for improvement in antigen-specific cancer immunotherapy of solid tumors. Adapted with permission from 91, copyright 2012 Springer Nature.

The direct delivery of peptide activators of CTLs into a tumor has a marked effect on tumor progression. Keman Cheng *et al*. developed a therapeutic peptide with the dual properties of tumor-targeting and on-demand-release of both DPPA-1, a peptide antagonist of programmed cell death-ligand 1, and NLG919, an inhibitor of indoleamine 2,3-dioxygenase. Using this therapeutic strategy, higher levels of tumor-infiltrated cytotoxic T cells were produced through the simultaneous suppression of immune checkpoints and tryptophan metabolism. The localized release of DPPA-1 and NLG919 increased the survival and activation of CTLs and resulted in the inhibition of melanoma growth (Figure 6A and B) 90. Daisuke Nobuoka *et al*. demonstrated that potent *in vitro* and *in vivo* cytotoxicity could be induced by human leukocyte antigen (HLA)-A*02:01-restricted glypican-3144-152 (FVGEFFTDV) and cytomegalovirus 495-503 (NLVPMVATV) peptide-specific CTLs. They also found that the direct injection of an ovalbumin 257-264 peptide (SIINFEKL) intratumorally effectively curbed the growth of ovalbumin-negative tumors and improved host survival without eliciting observable side effects (Figure 6C) 91.

Treg cells are regarded as a crucial mechanism of tumor immune escape and form a major barrier to the progress of many cancer immunotherapies. Lozano *et al*. found that a 15-mer synthetic peptide (P60) was able to bind to FOXP3, abrogate the FOXP3/AML1 interaction, and impede Treg cell activity, which caused significant anti-tumor activity both *in vitro* and *in vivo*. They further synthesized a macrocyclic peptide (P60-D2A-S5A) that had enhanced Treg cell inhibition and demonstrated that it could strengthen the anti-tumor activity of anti-PD1 antibodies against hepatocellular carcinoma 92.

### 3.2 B cells

**Figure 7:**
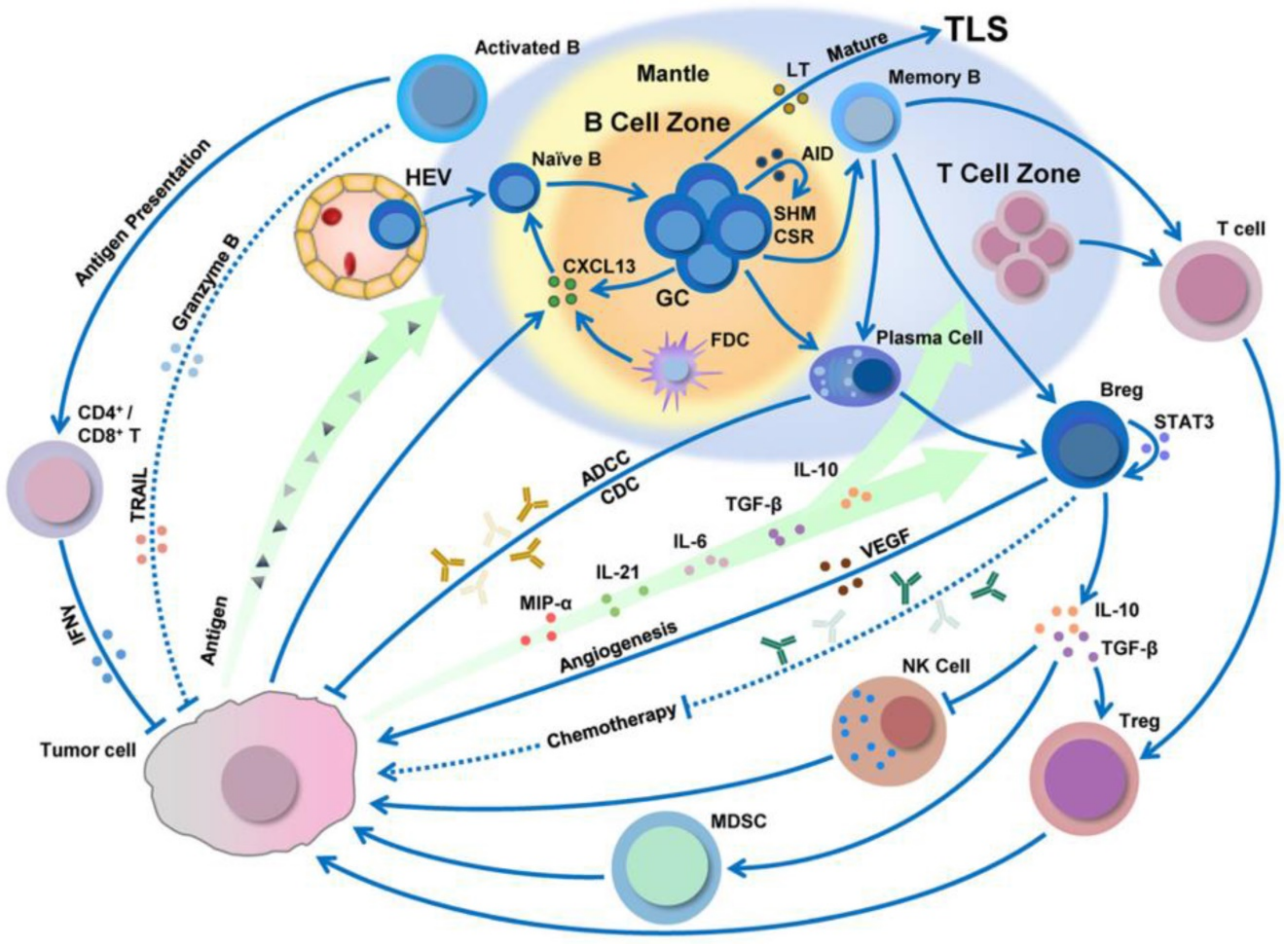
B cell infiltration, development, and polarization can be regulated by the tumor microenvironment. Adapted with permission from 94, copyright 2019 Springer Nature.

B cells are a lymphocyte subtype of white blood cells that play a significant role in the adaptive immune system by secreting antibodies. The antibodies generated by B cells have a high affinity and are functionally versatile, but their production requires a significant amount of time after exposure to antigens. On the other hand, B cells can respond more rapidly to T cell-dependent antigens, but they require T-cells to help activate B cells, the antibodies have lower affinity, and the antibodies are less functionally versatile. Besides this, tumor antigens can be processed by B cells and then presented to CD8^+^/CD4^+^ T cells (Figure 7) 93, 94.

Cancer vaccines based on B cell peptides are generally composed of an adjuvant and an immunogenic protein containing a B cell epitope peptide that can induce B cells to create antibodies. The antibodies produced with these vaccines are polyclonal mixtures that recognize a variety of antigens and differ from monoclonal antibodies with specific targeting sites. Another vaccine method is the use of B cell peptide mimics that can directly bind to tumor-specific cellular receptors and thereby prevent the dimerization of receptors. Furthermore, the mechanism of anti-tumor activity for antibodies or their peptide mimics is that they initially bind to the tumor receptors, block downstream signals, and induce several anti-tumor effect 95.

Several groups have proposed advanced strategies for the use of effective B cell vaccines as HER2-positive cancer immunotherapies. Clinical monoclonal antibodies (Trastuzumab and Pertuzumab) can specifically bind to B cell epitopes and elicit a promising polyclonal antibody response to HER2/neu in both preclinical and Phase I studies. Based on this success, HER-Vaxx and B-Vaxx were developed by Ursula Wiedermann and Pravin Kaumaya *et al.*, respectively, to treat tumors that overexpress the HER2/neu receptors 96, 97. HER-Vaxx is a HER2 multi-peptide vaccine with 3 HER2 peptides representing B cell epitopes that can produce specific IgG antibodies and has demonstrated strong antitumor activity in mice 96. This multi-peptide vaccine also restrained tumor immunological tolerance and yielded a similar effect to that observed with current clinical anti-HER2-based antibody therapies 98. KEY-Vaxx, another B cell vaccine developed by Pravin Kaumaya *et al*., also aimed to induce a polyclonal antibody response that could inhibit PD-1 signaling. Promisingly, KEY-Vaxx elicited significantly better antitumor effects in a mouse colorectal cancer preclinical study than a commercial anti-PD-1 antibody 99. A special HER2 peptide with epitopes from CD4^+^/CD8^+^ T-cells was developed by Miyako *et al*. that significantly delayed tumor growth 100.

In addition to the B cell-based peptide cancer vaccines that target PD-1 and HER2, peptide mimics that can inhibit EGFR signaling have also been attempted. Using a mimotype approach, epitope-specific immunization has produced an effective anti-EGFR immunotherapy that can elicit the production of “cetuximab-like” antibodies *in vivo* 101. Lei Zhu *et al*. synthesized peptides composed of a linear B cell epitope peptide from human EGFR and a Th-cell epitope to successfully target the dimer interface of EGFR. These peptides were highly immunogenic, stimulated high production of antibodies in animal models, and significantly inhibited tumor growth in patients 102. Collectively, this EGFR vaccine represents as a promising candidate for an effective anti-EGFR immunotherapy.

## 4. Peptide-based cancer vaccine

The goal of a cancer vaccine is to activate a patient's immune system and prime it to recognize and kill cancer cells. The vaccine should activate a patient's professional APCs, which are mainly DCs. After DCs have taken up and processed the antigens introduced by the vaccine, DCs transit to lymph nodes and expose the antigen on their cellular surface* via* MHC-I/II to activate T cells. As T cells are activated, they proliferate and further differentiate into CD8^+^ cytotoxic and CD4^+^ helper T cells. These cells can then target special antigens on the tumor cell surface after leaving lymph nodes and begin to destroy the tumor 103.

Peptide vaccines are usually made up of peptides and adjuvants 104, 105. Typical peptide vaccine strategies are being used to design personalized vaccines with synthetic peptides based upon tumor-specific antigens. Immune cell-specific vaccines activate the immune system to create polyclonal antibodies that have several advantages compared to synthetic monoclonal antibodies. These include a short production time, low cost, highly stability, high antibody affinity against the antigen based on recognition of multiple epitopes, and a better ability to capture the target protein 106, 107.

### 4.1 Peptide antigen vaccine

Peptide immunogenicity is correlated with a number of factors, including composition, length, and administration route. The adjustment of a peptide's composition with oxadiazole, tetrazole, and oxazole functionalities can aid in rigidification of peptide skeletons, which along with cyclization can increase the peptide's binding affinity. Bioavailability can also be adjusted by tailoring the peptide sequence to the proper environment 108. Short peptides are preferred because they can adopt similar conformations to those found with the native antigen. In addition, vaccines administered *via* intravenous injection (i.v.) can induce stronger CTL responses compared to responses caused by subcutaneous (s.c.) or intramuscular (i.m.) injections 109.

**Figure 8:**
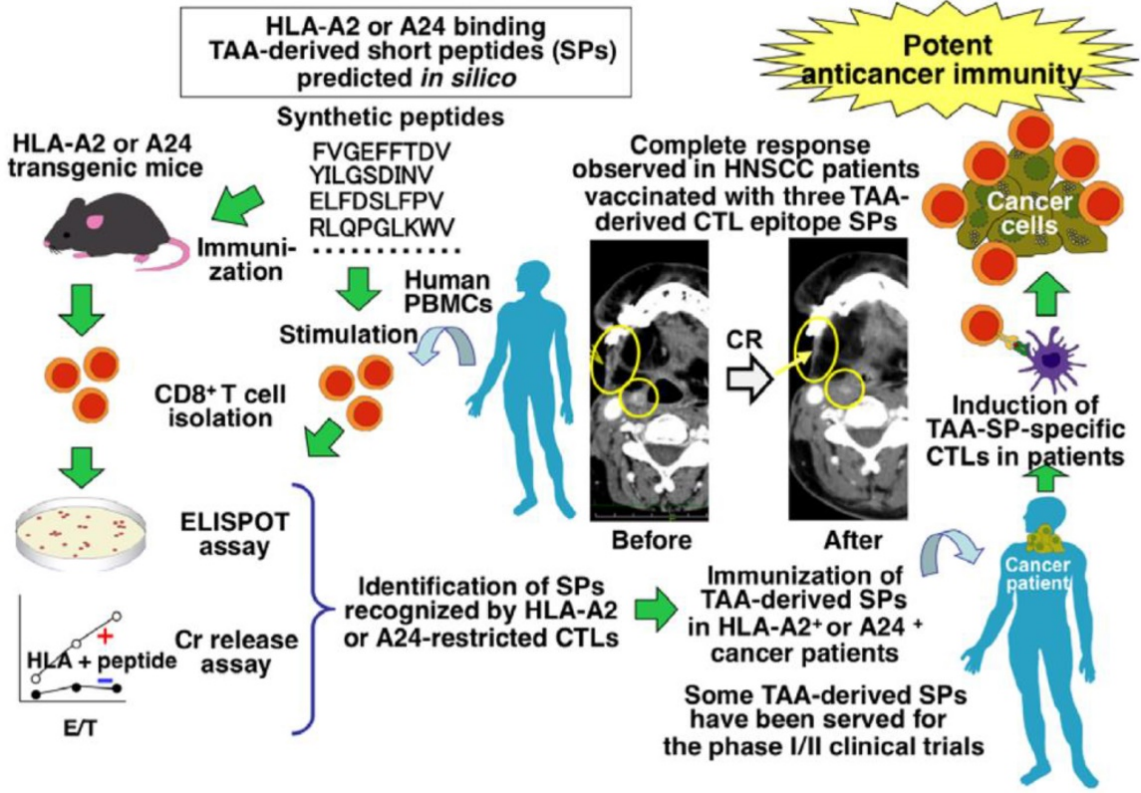
Identification of tumor-associated antigen (TAA)-derived short peptides recognized by CTLs and their application to cancer immunotherapy. Adapted with permission 110, copyright 2015 Wiley-VCH.

Peptide-based cancer vaccines are generally designed to target the activation of a specific effector cell type 111. Several peptide-based cancer vaccines in clinical phase I and II trials have chosen specific T-helper cell epitopes, TAA-derived CTL epitopes, or dendritic cells with TAA-derived peptides that provide practical clinical benefits to a small number of patients. MHC I peptides are shorter (8-10 amino acids) and can be recognized by CD8^+^ T cells, while MHC II peptides are longer (often 13 to 18 amino acids) and are generally recognized by CD4^+^ T cells 112. Martijn S. Bijker *et al*. have revealed that long peptides could promote higher quality T-cell responses than shorter versions, which generally were unable to kill cancer cells unless a longer MHC II peptide was added 113. The first long-peptide vaccination in humans was derived from two self-antigens, mucin and HER-2/neu, and demonstrated the safety and antigen specific T-cell responses that could be achieved with a synthetic peptide 17, 114. These initial clinical studies have shown that long peptides can be used to achieve more effective cancer immunotherapy vaccines than earlier attempts. Long peptides have the potential to elicit a significantly improved therapeutic effect, expand the T cell population in tumors, and increase the long-term memory of the immune system. However, the immunogenicity of peptide-based vaccines remains weak, which has highlighted the importance of combining of peptides with potent adjuvants to improve anti-tumor immune responses 115, 116. To improve the immunotherapeutic effect of peptide-based cancer vaccines, a clinical application program has been developed to combine immunotherapies with specific immune checkpoint targeting monoclonal antibody therapies like those targeting PD-1, PD-L1 and CTLA-4. Recent advances in genetic and bioinformatic analysis techniques have also improved the authentication of neoantigens (Figure 8) 110.

The identification of the specific peptide compositions and sequences of many cancer antigens has enabled the design of more targeted peptide-based cancer immunotherapies. An immunodominant synthetic peptide with increased HLA-A2 binding capabilities was designed by Steven A. Rosenberg *et al*. based on a melanoma-associated antigen sequence. This peptide was used as a vaccine for the treatment of metastatic melanoma and more than 90% of patients who received it achieved an immunized effect 117. Athanasios Kotsakis *et al*. demonstrated that a hTERT-targeting Vx-001 vaccine, which included a TERT572Y cryptic peptide, improved clinical immune responses in the majority of NSCLC HLA-A2^+^ patients 118. A peptide inhibitor identified by Alexander B. Sigalov *et al*. could specifically silence the TREM-1 receptor and significantly slowed the growth rate of NSCLC tumors 119. Aarif Ahsan *et al*. established that a synthetic peptide called disruptin (SVDNPHVC) targets EGFR-positive cancer and could not only reduce the clonogenicity of tumor cells, but also decreased the micro-vessel density in lung cancer tumors 120. By loading a Wilm's tumor-1 (WT1) peptide into a DC vaccine, Hidenori Takahashi *et al*. demonstrated that they could significantly improve the survival of patients with advanced NSCLC 121. Sumiyuki Nishida *et al*. reported that there was a close relationship for clinical delayed-type hypersensitivity (DTH)-positive patients between longer survival and a positive delayed-type hypersensitivity to WT1 peptides (CYTWNQMNL) and memory-phenotype WT1-specific CTLs 122. In addition, the combination of a WT1 peptide vaccine based on these peptides and gemcitabine was more effective than gemcitabine alone against advanced pancreatic cancer 122. Nobuhisa Ishikawa *et al*. reported a LY6K-177 peptide vaccine, based on the LY6K-177 peptide that is overexpressed in a majority of lung and esophageal cancers, could induce a specific CD8^+^ CTL response and significantly enhanced the survival rate of patients 123. A personalized peptide vaccination strategy was identified by Ryuji Takahashi *et al*. which achieved positive results for metastatic, recurrent triple-negative breast cancer patients in a Phase II clinical trial 124. Junya Ohtake *et al*. conjugated a synthetic long peptide with survivin to induce an IFN-γ response and thereby promote the differentiation of Th1 and Tc1 cells *in vivo* 125. While many of the current clinical tumor-associated antigen peptides have a great deal of promise, they are mainly recognized by CD8^+^ T cells and the variety amongst these peptides is very limited 126.

### 4.2 Other personalized anti-cancer vaccines

Recently, a number of other materials-based vaccine strategies have been developed. These include polymer scaffolds that direct immune cell function and micro- or nanoparticles that target special organs and cells. Tyrel Smith *et al*. fabricated an implantable biopolymer device using a collagen-mimetic peptide (GFO-GER) to deliver CAR T cells to the tumor 127. After injection at the tumor site, the device caused a considerable increase in the number of T cells in the tumor and was capable of eradicating tumors in both an orthotopic pancreatic cancer and a melanoma mouse model. Personalized vaccination against each patient's tumor-specific neoantigens by utilizing the unique mutation profile of each individual's tumor could greatly improve therapeutic outcomes 128. To increase the immunogenicity of a neoantigen-based vaccine, several novel vaccine delivery strategies including artificial scaffolds, have been designed and developed to mimic the target neoantigens' protein fold performance. Three important TLR-2 ligands (lipid core peptide, Pam2Cys and Pam3Cys) were conjugated to an engineered polytope that could self-assemble into nanoparticles and they were able to create higher antigen-specific IgG antibodies after vaccination in mice. Recently, Mooney *et al*. described a vaccine approach that used an injectable scaffold loaded with a selection of tumor-expressed peptides. The team made natural antigen-presenting cell-mimetic scaffolds (APC-ms) comprised of supported lipid bilayers formed on high-aspect-ratio mesoporous silica micro-rods that could readily absorb multiple peptides. They demonstrated that APC-ms promoted a 2 to 10-fold expansion of highly functional T cells for adoptive cell transfer compared with the conventional expansion systems 129.

Optimized nano-vaccines can more efficiently deliver many types of antigens, adjuvants, and immune regulatory agents than more traditional vaccination methods 130-132. Self-assembling peptides are considered promising agents for immunotherapies because they can induce strong immune responses in the absence of supplemental adjuvants. Fiber-forming peptides like Q11, KFE8, and RADA can form cylindrical micelles that can be functionalized as an adjuvant for T or B cells. Peptides carrying multiple epitopes can cause significant immune responses without significant inflammation and have received extensive attention as the next generation of cancer treatments. Collier* et al*. reported that an epitope-bearing peptide could self-assemble into elongated peptide nanofibers with α-helical structures that were easily internalized by APCs. These nanofibers were able to induce responses in CD_4_^+^ T cells and CD_8_^+^ T cells in the absence of adjuvants *in vivo* 133.

Currently, peptide materials that induce a strong anti-tumor immuno-response have been used as potential cancer vaccines. TAAs are a common target of peptide-based vaccines that are capable of being identified by the immune systems and these vaccines can cause cancer cell disruption and tumor extinction 126. However, vaccines based on peptide materials also have displayed poor clinical efficacy due to the poor immunogenicity of TAAs, immune escape by tumor cells, and tumor heterogeneity 134. To improve the clinical effects for cancer patients, some new approaches like personalized peptide vaccination 135, identifying novel TAA-based peptide materials 136, designing peptide-based vaccines with multiple TAA epitopes 137, and the combination of peptide-based vaccines with chemotherapies need to be urgently developed 138 .

## 5. Immunomodulating peptide delivery systems

**Figure 9:**
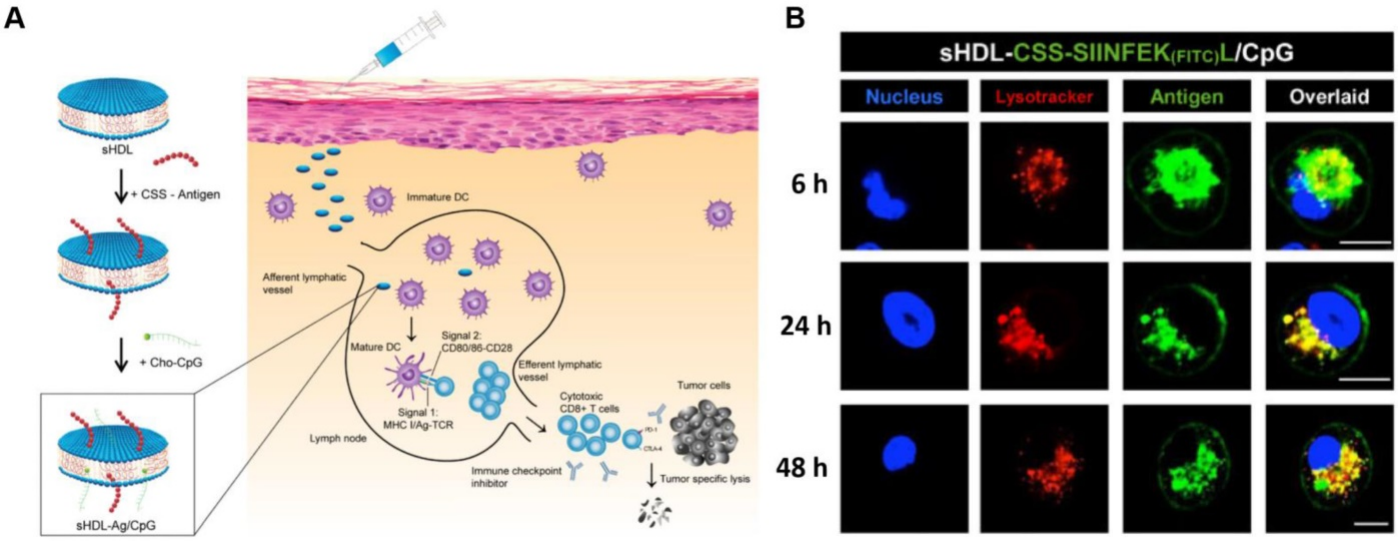
sHDL nanodisc cancer vaccine strategy and *in vitro* results. **A)** Design of sHDL nanodisc platform for personalized cancer vaccines. **B)** Strong and durable antigen peptide presentation mediated by sHDL nanodiscs. JAWSII cells were incubated sHDL-CSSSIINFEK(FITC)L/CpG for 6, 24, or 48 h, followed by staining with Hoechst and Lysotracker. Adapted with permission from 15, copyright 2017 Springer Nature.

The nanostructures of therapeutic peptides not only provide stable spatial conformations and special cancer targeting abilities, but they can also overcome the pharmaceutical obstacles inherent in peptides. A major hurdle to the development of successful and effective vaccines is the design of antigen delivery systems that optimize antigen presentation and induce key protective immune responses. The use of a nanomaterial-based delivery system should be able to better achieve such effects than conventional methods. A nanomaterial-based approach should enhance the targeting of vaccines, trigger better immune responses, increase the cellular uptake of vaccines, increase the vaccine's ability to pass through biological barriers, and minimize a treatment's side effects 139. The composition, charge, and size of nanoparticles can be used to modulate the cellular uptake and biodistribution of vaccines* in vivo* after injection 115, 140. The shape and flexibility of nanoparticles can have a particular impact on their biological fate as it has been reported that rod-like particles can be taken up more efficiently by HeLa cells when compared with spheres, cylinders, and cubes. The proper design could also significantly improve bioavailability *in vivo.* Feng Qiu *et al*. described a pH-responsive nanoparticle assembled from antigenic peptides and poly(propylacrylic acid) with endosomal escape activity. This nanoparticle was able to stimulate CD8^+^ T cell activity by increasing antigen uptake and presentation on DC MHC-I molecules. The combination of this nanoparticle with α-galactosylceramide (an immune adjuvant) could induce strong CD8^+^ T cell responses and prolong survival time in mice bearing melanoma tumors 141. Jihua Hao *et al*. developed a micellar vaccine based on a model peptide antigen with four cysteines to create disulfide linkages that stabilized the micellular structure. After phagocytosis of the micelle by DCs, the peptide would be released from micelles as the disulfide bonds were broken within endosomes. This release both improved the concentration of the peptide within DCs and enhanced its tumor immune response 142. A synthetic lipoprotein nanodisc-based platform (sHDL) consisting of peptide antigens and adjuvants was developed by Rui Kuai *et al*. that displayed enhanced accumulation in lymphoid organs and improved antigen presentation. Interestingly, this nano-platform based vaccine induced a 47-fold increase in neoantigen-specific CTLs compared to other soluble vaccines (Figure 9) 15. Peipei Zhang *et al*. developed a nanoparticle-coated polyelectrolyte multilayer on gold nanoparticle templates and then loaded special peptide antigens and polyanionic TLRs that could be activated after being internalized by DCs. These nanoparticles could trigger special TLR signals and lead to the presentation of peptide antigens. They also enhanced the proliferation of antigen-specific T-cells and increased the concentration of antigen-specific CD8^+^ T cells in peripheral blood 143. An mRNA delivery nano-system was developed by Anne-Line Coolen *et al*. that incorporated a poly(lactic acid) nanoparticle and a cationic cell-penetrating peptide. This nanoparticle induced enhanced protein expression within DCs *in vitro* and could activate endosomal and cytosolic pattern recognition receptors that could be used to tune the DC-based innate immune response 144.

**Figure 10:**
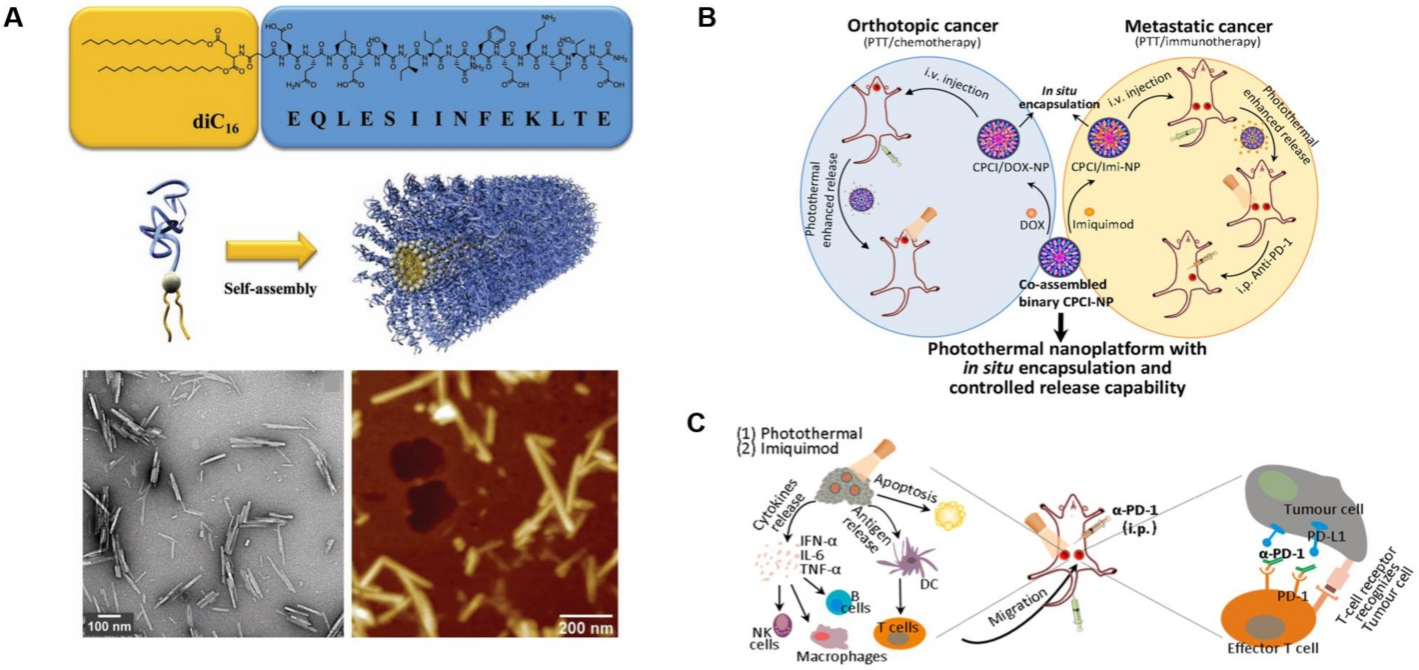
Design and anticancer activity of a photothermal immuno-nanoplatform. **A)** Peptide amphiphile and micelle structure. TEM and AFM of micelles in PBS reveals cylindrical micelles. Adapted with permission from 148, copyright 2012 WILEY‐VCH. **B)** Illustration of the *in situ* encapsulation and controlled release capability of cytotoxic agents and immunomodulatory agents against orthotopic oral cancer and metastatic breast cancer. **C)** Schematic illustration of the proposed mechanism of antitumor immune responses induced by the nanoparticle in combination with anti-PD-1 therapy. Adapted with permission from 149, copyright 2018 American Chemical Society.

There are 11 FDA-approved slow-release microsphere injections on the market at present, such as PEG 3350, the commercial drug Eligard® Leuprolide (composed of PLGA and Leuprolide acetate), and Janssen Pharmaceuticals' PLG-containing Risperdal Consta® 145, 146. The success of these microsphere-based therapies has increased the focus on developing novel techniques in this area. Zhiping Zhang *et al*. created a biodegradable nanoparticle with poly(d,l-lactide-*co*-glycolide) and two antigenic peptides derived from murine melanoma, TRP2_180-188_ (SVYDFFVWL) and hgp100_25-33_ (KVPRNQDWL). These nanoparticles showed excellent uptake, strong antigen-specific T cell responses, and significant antitumor activity 147. A peptide amphiphile was developed by Matthew Black *et al*. that linked a model cytotoxic T-cell epitope to a synthetic lipid (diC16-EQLESIINFEKLTE). The amphiphile can assemble into a cylindrical nanoparticle, induce enhanced cytotoxic T-cell responses, and significantly inhibit tumor growth (Figure 10A) 148. Lu Zhang *et al*. reported a photothermal immuno-nanoplatform based on peptide materials that displayed a positive inhibitory effect on 4T1 syngeneic murine breast tumors after combining them with imiquimod and an anti-PD-1 antibody. This therapeutic strategy eradicated both primary and distant tumors via photothermal and immune effects and improved the immune-response rate in patients (Figure 10B and C) 149.

**Figure 11:**
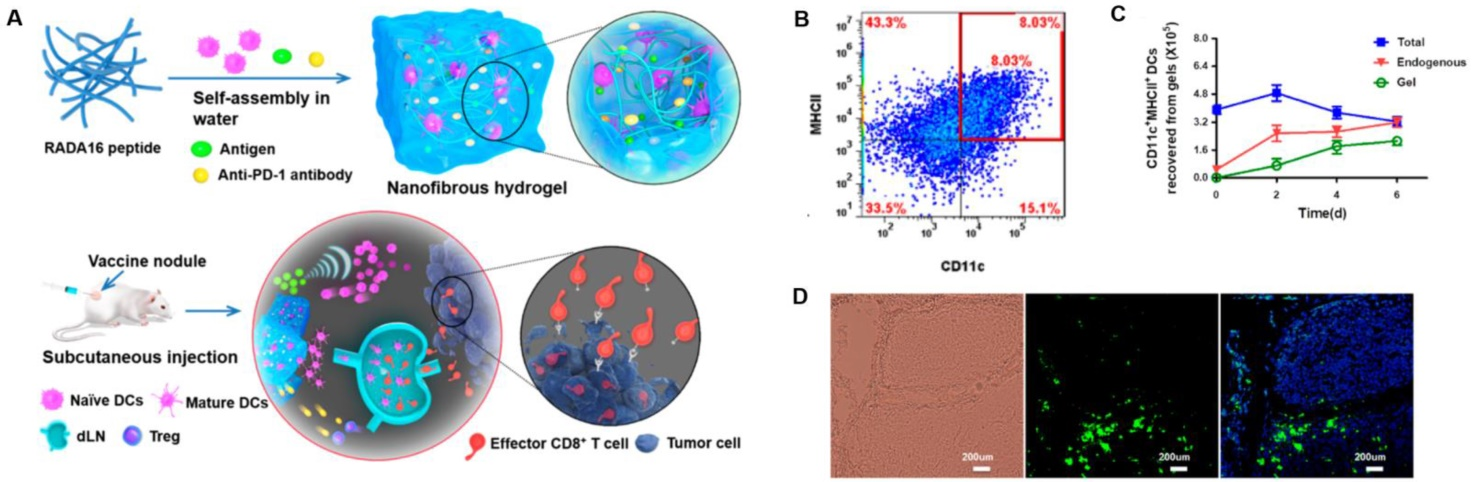
Peptide-based nanogels and their use as immunologic agents against cancer. **A)** Formation and the mechanism of action for the DC-based vaccine nodule engineered in a nanofibrous peptide hydrogel. Delivery of exogenous DCs and recruitment of host DCs *in vivo* by the vaccine nodule. **B)** Representative scatter plots illustrating the identification of MHCII+CD11c+ DCs (in red rectangle) recovered from gels and **C)** quantification of MHCII+CD11c+ DCs in gels at various time points. **D)** Representative optical sectioning of dLNs explanted at day 1 after injection. DCs in the dLNs are indicated by GFP (green), and the nuclei of T cells were stained by DAPI (blue). Adapted with permission from 51, copyright 2018 American Chemical Society.

Peptide-based nanogels are also commonly used for the delivery of therapeutic agents. Daisuke Muraoka *et al*. reported a cholesteryl pullulan-based nanogel encapsulating a synthetic long special-antigen peptide that effectively delayed tumor growth. This study also implied that the strong stimulation response of lymph node macrophages to TLR was a basis for the efficacy of macrophage-guided nanogel vaccines 150. Pengxiang Yang *et al*. designed a peptide-based nanofibrous hydrogel vaccine that incorporated an anti-PD-1 antibody, DCs, and a tumor antigen. The hydrogel vaccine could effectively stimulate anti-tumor T-cell immunity and significantly enhanced the activation/infiltration of CD8^+^ effector T-cell in tumor tissue. This gave the nanoparticle potent tumor growth inhibition and prolonged the survival of mice in prophylactic/therapeutic tumor models (Figure 11) 51.

## 6. Summary

In this review, we have illustrated the key roles of different immune cells and summarized the applications of peptide materials in both the innate and adaptive immune systems. The design, categories, and applications of peptide-based anti-cancer materials were also comprehensively covered. Cancer vaccinations based on peptide materials can significantly enhance systemic immune responses, generate effective immune cell responses in tumor tissue, and inhibit malignant tumor metastasis/recurrence. Peptide-based delivery systems that enable the delivery of therapeutic cargoes into specific cell lines with low toxicity and high therapeutic effect are fundamentally important for peptide cancer vaccines. The majorities of peptide therapeutics are effectively administered by parenteral routes in clinical trials but are generally less suitable for oral administration. Improvements in the stability and permeability of formulated peptide drugs or vaccines may also facilitate the availability of peptide materials in clinical cancer immunotherapies. Noteworthy advances in peptide screening and computational biology can also be applied to discover new categories of peptide-based formulations and advance current therapies. In addition, metabolomic, proteomic, and genomic screening of natural products can be used to identify bioactive peptides generated by uncommon post-translational modifications or non-ribosomal synthesis. Moreover, in order to be clinically translated, these peptide-based platforms must be stable for large-scale production and storage. With the consideration of the difficulty in studying the pharmacokinetics and biodistribution of peptides, exploration of the optimal doses and dosing intervals for a peptide could identify key parameters that determine their therapeutic effects. As the field of immuno-oncology improves, we sincerely expect the synergistic development of peptide materials with other immunotherapies to yield innovative strategies for tumor immunotherapy.

## References

[1] Rosenberg SA, Yang JC, Restifo NP (2004). Cancer immunotherapy: moving beyond current vaccines. Nat Med.

[2] Pardoll DM (2012). The blockade of immune checkpoints in cancer immunotherapy. Nat Rev Cancer.

[3] Song W, Musetti SN, Huang L (2017). Nanomaterials for cancer immunotherapy. Biomaterials.

[4] Sun H, Dong Y, Feijen J, Zhong Z (2018). Peptide-decorated polymeric nanomedicines for precision cancer therapy. J Control Release.

[5] Tang W, Becker ML (2014). “Click” reactions: a versatile toolbox for the synthesis of peptide-conjugates. Chem Soc Rev.

[6] Komin A, Russell L, Hristova KA, Searson P (2017). Peptide-based strategies for enhanced cell uptake, transcellular transport, and circulation: Mechanisms and challenges. Adv Drug Delivery Rev.

[7] Zhao J, Li Q, Hao X, Ren X, Guo J, Feng Y (2017). Multi-targeting peptides for gene carriers with high transfection efficiency. J Mater Chem B.

[8] Wei G, Wang Y, Huang X, Hou H, Zhou S (2018). Peptide-Based Nanocarriers for Cancer Therapy. Small Methods.

[9] Song H, Yang P, Huang P, Zhang C, Kong D, Wang W (2019). Injectable polypeptide hydrogel-based co-delivery of vaccine and immune checkpoint inhibitors improves tumor immunotherapy. Theranostics.

[10] Qin H, Ding Y, Mujeeb A, Zhao Y, Nie G (2017). Tumor microenvironment targeting and responsive peptide-based nanoformulations for improved tumor therapy. Mol Pharmacol.

[11] Laakkonen P, Porkka K, Hoffman JA, Ruoslahti E (2002). A tumor-homing peptide with a targeting specificity related to lymphatic vessels. Nat Med.

[12] Cieslewicz M, Tang J, Jonathan LY, Cao H, Zavaljevski M, Motoyama K (2013). Targeted delivery of proapoptotic peptides to tumor-associated macrophages improves survival. Proc Natl Acad Sci.

[13] Gautam A, Kapoor P, Chaudhary K, Kumar R, Raghava G, Consortium SDD (2014). Tumor homing peptides as molecular probes for cancer therapeutics, diagnostics and theranostics. Curr Med Chem.

[14] Porkka K, Laakkonen P, Hoffman JA, Bernasconi M, Ruoslahti E (2002). A fragment of the HMGN2 protein homes to the nuclei of tumor cells and tumor endothelial cells *in vivo*. Proc Natl Acad Sci.

[15] Kuai R, Ochyl LJ, Bahjat KS, Schwendeman A, Moon JJ (2017). Designer vaccine nanodiscs for personalized cancer immunotherapy. Nat Mater.

[16] Rosenberg SA, Yang JC, Schwartzentruber DJ, Hwu P, Marincola FM, Topalian SL (1998). Immunologic and therapeutic evaluation of a synthetic peptide vaccine for the treatment of patients with metastatic melanoma. Nat Med.

[17] Gjertsen MK, Breivik J, Saeterdal I, Thorsby E, Gaudernack G, Bakka A (1995). Vaccination with mutant ras peptides and induction of T-cell responsiveness in pancreatic carcinoma patients carrying the corresponding RAS mutation. Lancet.

[18] Lam KS, Salmon SE, Hersh EM, Hruby VJ, Kazmierski WM, Knapp RJ (1991). A new type of synthetic peptide library for identifying ligand-binding activity. Nature.

[19] Zhang D, Qi GB, Zhao YX, Qiao SL, Yang C, Wang H (2015). *In situ* formation of nanofibers from purpurin18-peptide conjugates and the assembly induced retention effect in tumor sites. Adv Mater.

[20] Wadia JS, Dowdy SF (2005). Transmembrane delivery of protein and peptide drugs by TAT-mediated transduction in the treatment of cancer. Adv Drug Delivery Rev.

[21] Bidwell III GL, Raucher D (2010). Cell penetrating elastin-like polypeptides for therapeutic peptide delivery. Adv Drug Delivery Rev.

[22] Rodriguez-Cabello JC, Arias FJ, Rodrigo MA, Girotti A (2016). Elastin-like polypeptides in drug delivery. Adv Drug Delivery Rev.

[23] Chen J, Yao Y, Gong C, Yu F, Su S, Chen J (2011). CCL18 from tumor-associated macrophages promotes breast cancer metastasis via PITPNM3. Cancer cell.

[24] Gordon S (2003). Alternative activation of macrophages. Nat Rev Immunol.

[25] Gordon S, Martinez FO (2010). Alternative activation of macrophages: mechanism and functions. Immunity.

[26] Martinez FO, Helming L, Gordon S (2009). Alternative activation of macrophages: an immunologic functional perspective. Annu Rev Immunol.

[27] Varin A, Gordon S (2009). Alternative activation of macrophages: immune function and cellular biology. Immunobiology.

[28] Gao S, Yang D, Fang Y, Lin X, Jin X, Wang Q (2019). Engineering Nanoparticles for Targeted Remodeling of the Tumor Microenvironment to Improve Cancer Immunotherapy. Theranostics.

[29] David A (2017). Peptide ligand-modified nanomedicines for targeting cells at the tumor microenvironment. Adv Drug Delivery Rev.

[30] Tang X, Mo C, Wang Y, Wei D, Xiao H (2013). Anti-tumour strategies aiming to target tumour-associated macrophages. Immunology.

[31] Gajewski TF, Woo S-R, Zha Y, Spaapen R, Zheng Y, Corrales L (2013). Cancer immunotherapy strategies based on overcoming barriers within the tumor microenvironment. Curr Opin Immunol.

[32] Conde J, Bao C, Tan Y, Cui D, Edelman ER, Azevedo HS (2015). Dual targeted immunotherapy via *in vivo* delivery of biohybrid RNAi-peptide nanoparticles to tumor-associated macrophages and cancer cells. Adv Funct Mater.

[33] Qian Y, Qiao S, Dai Y, Xu G, Dai B, Lu L (2017). Molecular-targeted immunotherapeutic strategy for melanoma via dual-targeting nanoparticles delivering small interfering RNA to tumor-associated macrophages. ACS Nano.

[34] Ngambenjawong C, Cieslewicz M, Schellinger JG, Pun SH (2016). Synthesis and evaluation of multivalent M2pep peptides for targeting alternatively activated M2 macrophages. J Control Release.

[35] Shen K-Y, Song Y-C, Chen I-H, Chong P, Liu S-J (2014). Depletion of tumor-associated macrophages enhances the anti-tumor immunity induced by a Toll-like receptor agonist-conjugated peptide. Hum Vaccines Immunother.

[36] Peng M, Zhang Q, Cheng Y, Fu S, Yang H, Guo X (2017). Apolipoprotein AI mimetic peptide 4F suppresses tumor-associated macrophages and pancreatic cancer progression. Oncotarget.

[37] Fang WB, Yao M, Brummer G, Acevedo D, Alhakamy N, Berkland C (2016). Targeted gene silencing of CCL2 inhibits triple negative breast cancer progression by blocking cancer stem cell renewal and M2 macrophage recruitment. Oncotarget.

[38] Rodriguez PL, Harada T, Christian DA, Pantano DA, Tsai RK, Discher DE (2013). Minimal" Self" peptides that inhibit phagocytic clearance and enhance delivery of nanoparticles. Science.

[39] Muller AJ, Scherle PA (2006). Targeting the mechanisms of tumoral immune tolerance with small-molecule inhibitors. Nat Rev Cancer.

[40] Kavanagh B, Ko A, Venook A, Margolin K, Zeh H, Lotze M (2007). Vaccination of metastatic colorectal cancer patients with matured dendritic cells loaded with multiple major histocompatibility complex class I peptides. J Immunother.

[41] Brinkman JA, Fausch SC, Weber JS, Kast WM (2004). Peptide-based vaccines for cancer immunotherapy. Expert Opin Biol Ther.

[42] Yan Z, Wu Y, Du J, Li G, Wang S, Cao W (2016). A novel peptide targeting Clec9a on dendritic cell for cancer immunotherapy. Oncotarget.

[43] Wang R-F, Wang HY (2002). Enhancement of antitumor immunity by prolonging antigen presentation on dendritic cells. Nat Biotechnol.

[44] Choi YJ, Park S-J, Park Y-S, Park HS, Yang KM, Heo K (2018). EpCAM peptide-primed dendritic cell vaccination confers significant anti-tumor immunity in hepatocellular carcinoma cells. PloS one.

[45] Ueda Y, Itoh T, Nukaya I, Kawashima I, Okugawa K, Yano Y (2004). Dendritic cell-based immunotherapy of cancer with carcinoembryonic antigen-derived, HLA-A24-restricted CTL epitope: Clinical outcomes of 18 patients with metastatic gastrointestinal or lung adenocarcinomas. Int J Oncol.

[46] Sadanaga N, Nagashima H, Mashino K, Tahara K, Yamaguchi H, Ohta M (2001). Dendritic cell vaccination with MAGE peptide is a novel therapeutic approach for gastrointestinal carcinomas. Clin Cancer Res.

[47] Vansteenkiste J, Zielinski M, Linder A, Dahabreh J, Gonzalez EE, Malinowski W (2013). Adjuvant MAGE-A3 immunotherapy in resected non-small-cell lung cancer: phase II randomized study results. J Clin Oncol.

[48] Vansteenkiste JF, Cho BC, Vanakesa T, De Pas T, Zielinski M, Kim MS (2016). Efficacy of the MAGE-A3 cancer immunotherapeutic as adjuvant therapy in patients with resected MAGE-A3-positive non-small-cell lung cancer (MAGRIT): a randomised, double-blind, placebo-controlled, phase 3 trial. Lancet Oncol.

[49] Zajac P, Schultz-Thater E, Tornillo L, Sadowski C, Trella E, Mengus C (2017). MAGE-A Antigens and Cancer Immunotherapy. Front Med.

[50] Fong L, Hou Y, Rivas A, Benike C, Yuen A, Fisher GA (2001). Altered peptide ligand vaccination with Flt3 ligand expanded dendritic cells for tumor immunotherapy. Proc Natl Acad Sci.

[51] Yang P, Song H, Qin Y, Huang P, Zhang C, Kong D (2018). Engineering dendritic-cell-based vaccines and PD-1 blockade in self-assembled peptide nanofibrous hydrogel to amplify antitumor T-cell immunity. Nano Lett.

[52] Patel S, Fu S, Mastio J, Dominguez GA, Purohit A, Kossenkov A (2018). Unique pattern of neutrophil migration and function during tumor progression. Nat Immunol.

[53] Smith JA (1994). Neutrophils, host defense, and inflammation: a double-edged sword. J Leukocyte Biol.

[54] Pham CT (2006). Neutrophil serine proteases: specific regulators of inflammation. Nat Rev Immunol.

[55] Nathan C (2006). Neutrophils and immunity: challenges and opportunities. Nat Rev Immunol.

[56] Gregory AD, Houghton AM (2011). Tumor-associated neutrophils: new targets for cancer therapy. Cancer Res.

[57] Mantovani A, Cassatella MA, Costantini C, Jaillon S (2011). Neutrophils in the activation and regulation of innate and adaptive immunity. Nat Rev Immunol.

[58] Shaul ME, Fridlender ZG (2019). Tumour-associated neutrophils in patients with cancer.

[59] Fridlender ZG, Sun J, Kim S, Kapoor V, Cheng G, Ling L (2009). Polarization of tumor-associated neutrophil phenotype by TGF-β:“N1” versus “N2” TAN. Cancer cell.

[60] Liu G, Shi H, Xie Z, Shen H, Huang H, Deng J (2009). Epithelial neutrophil-activating peptide-78 recruits neutrophils into pleural effusion. Eur Respir J.

[61] Kerros C, Tripathi SC, Zha D, Mehrens JM, Sergeeva A, Philips AV (2017). Neuropilin-1 mediates neutrophil elastase uptake and cross-presentation in breast cancer cells. J Biol Chem.

[62] Mazzucchelli L, Burritt JB, Jesaitis AJ, Nusrat A, Liang TW, Gewirtz AT (1999). Cell-specific peptide binding by human neutrophils. Blood.

[63] Miettinen HM, Gripentrog JM, Lord CI, Nagy JO (2018). CD177-mediated nanoparticle targeting of human and mouse neutrophils. PloS one.

[64] Albrethsen J, Bøgebo R, Gammeltoft S, Olsen J, Winther B, Raskov H (2005). Upregulated expression of human neutrophil peptides 1, 2 and 3 (HNP 1-3) in colon cancer serum and tumours: a biomarker study. BMC Cancer.

[65] Gaspar D, Freire JM, Pacheco TR, Barata JT, Castanho MA (2015). Apoptotic human neutrophil peptide-1 anti-tumor activity revealed by cellular biomechanics. Biochim Biophys Acta, Mol Cell Res.

[66] Ibrahim SA, Kulshrestha A, Katara GK, Amin MA, Beaman KD (2016). Cancer derived peptide of vacuolar ATPase 'a2'isoform promotes neutrophil migration by autocrine secretion of IL-8. Sc Rep.

[67] Caligiuri MA (2008). Human natural killer cells. Blood.

[68] Das J, Khakoo SI (2015). NK cells: tuned by peptide?. Immunol Rev.

[69] Guillerey C, Huntington ND, Smyth MJ (2016). Targeting natural killer cells in cancer immunotherapy. Nat Immunol.

[70] Chapel A, Garcia-Beltran WF, Hölzemer A, Ziegler M, Lunemann S, Martrus G (2017). Peptide-specific engagement of the activating NK cell receptor KIR2DS1. Sci Rep.

[71] Khakoo SI, Cassidy S, Cheent K (2014). Effects of Peptide on NK cell-mediated MHC I recognition. Front Immunol.

[72] Cheent KS, Jamil KM, Cassidy S, Liu M, Mbiribindi B, Mulder A (2013). Synergistic inhibition of natural killer cells by the nonsignaling molecule CD94. Proc Natl Acad Sci.

[73] Hammer Q, Rückert T, Borst EM, Dunst J, Haubner A, Durek P (2018). Peptide-specific recognition of human cytomegalovirus strains controls adaptive natural killer cells. Nat Immunol.

[74] Arina A, Murillo O, Dubrot J, Azpilikueta A, Alfaro C, Perez-Gracia JL (2007). Cellular liaisons of natural killer lymphocytes in immunology and immunotherapy of cancer. Expert Opin Biol Ther.

[75] Watzl C (2014). How to trigger a killer: modulation of natural killer cell reactivity on many levels.

[76] Mellman I, Coukos G, Dranoff G (2011). Cancer immunotherapy comes of age. Nature.

[77] Cha E, Klinger M, Hou Y, Cummings C, Ribas A, Faham M (2014). Improved survival with T cell clonotype stability after anti-CTLA-4 treatment in cancer patients. Sci Transl Med.

[78] Janeway Jr CA, Travers P, Walport M, Shlomchik MJ (2001). Principles of innate and adaptive immunity. Immunobiology: The Immune System in Health and Disease.

[79] Baitsch L, Baumgaertner P, Devêvre E, Raghav SK, Legat A, Barba L (2011). Exhaustion of tumor-specific CD8+ T cells in metastases from melanoma patients. J Clin Invest.

[80] Thomas DA, Massagué J (2005). TGF-β directly targets cytotoxic T cell functions during tumor evasion of immune surveillance. Cancer Cell.

[81] Desrichard A, Snyder A, Chan TA (2016). Cancer neoantigens and applications for immunotherapy. Clin Cancer Res.

[82] Postow MA, Chesney J, Pavlick AC, Robert C, Grossmann K, McDermott D (2015). Nivolumab and ipilimumab versus ipilimumab in untreated melanoma. N Engl J Med.

[83] Le DT, Uram JN, Wang H, Bartlett BR, Kemberling H, Eyring AD (2015). PD-1 blockade in tumors with mismatch-repair deficiency. N Engl J Med.

[84] Brahmer J, Reckamp KL, Baas P, Crinò L, Eberhardt WE, Poddubskaya E (2015). Nivolumab versus docetaxel in advanced squamous-cell non-small-cell lung cancer. New Engl J Med.

[85] Schwartzentruber DJ, Lawson DH, Richards JM, Conry RM, Miller DM, Treisman J (2011). gp100 peptide vaccine and interleukin-2 in patients with advanced melanoma. New Engl J Med.

[86] Robert C, Schachter J, Long GV, Arance A, Grob JJ, Mortier L (2015). Pembrolizumab versus ipilimumab in advanced melanoma. New Engl J Med.

[87] Hamid O, Robert C, Daud A, Hodi FS, Hwu W-J, Kefford R (2013). Safety and tumor responses with lambrolizumab (anti-PD-1) in melanoma. New Engl J Med.

[88] Li C, Zhang N, Zhou J, Ding C, Jin Y, Cui X (2018). Peptide blocking of PD-1/PD-L1 interaction for cancer immunotherapy. Cancer Immunol Res.

[89] Chang HN, Liu BY, Qi YK, Zhou Y, Chen YP, Pan KM (2015). Blocking of the PD-1/PD-L1 Interaction by ad-Peptide Antagonist for Cancer Immunotherapy. Angew Chem Int Ed.

[90] Cheng K, Ding Y, Zhao Y, Ye S, Zhao X, Zhang Y (2018). Sequentially responsive therapeutic peptide assembling nanoparticles for dual-targeted cancer immunotherapy. Nano Lett.

[91] Nobuoka D, Yoshikawa T, Takahashi M, Iwama T, Horie K, Shimomura M (2013). Intratumoral peptide injection enhances tumor cell antigenicity recognized by cytotoxic T lymphocytes: a potential option for improvement in antigen-specific cancer immunotherapy. Cancer Immunol Immunother.

[92] Lozano T, Gorraiz M, Lasarte-Cia A, Ruiz M, Rabal O, Oyarzabal J (2017). Blockage of FOXP3 transcription factor dimerization and FOXP3/AML1 interaction inhibits T regulatory cell activity: sequence optimization of a peptide inhibitor. Oncotarget.

[93] Yuen GJ, Demissie E, Pillai S (2016). B lymphocytes and cancer: a love-hate relationship. Trends Cancer.

[94] Wang S-s, Liu W, Ly D, Xu H, Qu L, Zhang L (2018). Tumor-infiltrating B cells: their role and application in anti-tumor immunity in lung cancer. Cell Mol Immunol.

[95] Kaumaya PT (2015). A paradigm shift: Cancer therapy with peptide-based B-cell epitopes and peptide immunotherapeutics targeting multiple solid tumor types: Emerging concepts and validation of combination immunotherapy. Hum Vaccines Immunother.

[96] Wiedermann U, Wiltschke C, Jasinska J, Kundi M, Zurbriggen R, Garner-Spitzer E (2010). A virosomal formulated Her-2/neu multi-peptide vaccine induces Her-2/neu-specific immune responses in patients with metastatic breast cancer: a phase I study. Breast Cancer Res Treat.

[97] Dakappagari NK, Douglas DB, Triozzi PL, Stevens VC, Kaumaya PT (2000). Prevention of mammary tumors with a chimeric HER-2 B-cell epitope peptide vaccine. Cancer research.

[98] Wiedermann U, Davis AB, Zielinski CC (2013). Vaccination for the prevention and treatment of breast cancer with special focus on Her-2/neu peptide vaccines. Breast Cancer Res Treat.

[99] Bekaii-Saab T, Overholser J, Yang Y, Penichet M, Kaumaya P (2019). Development of a novel PD-1 vaccine and in combination with two Chimeric HER-2 peptide vaccine provides synergistic inhibition of tumor growth in a syngeneic Balb/c model challenged with CT26/HER-2 carcinoma cell line.

[100] Miyako H, Kametani Y, Katano I, Ito R, Tsuda B, Furukawa A (2011). Antitumor effect of new HER2 peptide vaccination based on B cell epitope. Anticancer Res.

[101] Riemer AB, Kurz H, Klinger M, Scheiner O, Zielinski CC, Jensen-Jarolim E (2005). Vaccination With Cetuximab Mimotopes and Biological Properties of Induced Anti-Epidermal Growth Factor Receptor Antibodies. J Natl Cancer Inst.

[102] Zhu L, Zhao L, Wu M, Chen Z, Li H (2013). B-cell epitope peptide vaccination targeting dimer interface of epidermal growth factor receptor (EGFR). Immunol Lett.

[103] Kastenmüller W, Kastenmüller K, Kurts C, Seder RA (2014). Dendritic cell-targeted vaccines—hope or hype?. Nat Rev Immunol.

[104] Drake CG, Lipson EJ, Brahmer JR (2014). Breathing new life into immunotherapy: review of melanoma, lung and kidney cancer. Nat Rev Clin Oncol.

[105] Liu L, Chen Q, Ruan C, Chen X, Zhang Y, He X (2018). Platinum-Based Nanovectors Engineered with Immuno-Modulating Adjuvant for Inhibiting Tumor growth and Promoting Immunity. Theranostics.

[106] Lipman NS, Jackson LR, Trudel LJ, Weis-Garcia F (2005). Monoclonal versus polyclonal antibodies: distinguishing characteristics, applications, and information resources. ILAR J.

[107] Tolstrup AB, Frandsen TP, Bregenholt S (2006). Development of recombinant human polyclonal antibodies for the treatment of complex human diseases. Expert Opin Biol Ther.

[108] Meister D, Taimoory SM, Trant JF (2019). Unnatural amino acids improve affinity and modulate immunogenicity: Developing peptides to treat MHC type II autoimmune disorders. Pept Sci.

[109] Sultan H, Kumai T, Nagato T, Wu J, Salazar AM, Celis E (2019). The route of administration dictates the immunogenicity of peptide-based cancer vaccines in mice.

[110] Nishimura Y, Tomita Y, Yuno A, Yoshitake Y, Shinohara M (2015). Cancer immunotherapy using novel tumor-associated antigenic peptides identified by genome-wide cDNA microarray analyses. Cancer Sci.

[111] Xiao Y-F, Jie M-M, Li B-S, Hu C-J, Xie R, Tang B (2015). Peptide-based treatment: a promising cancer therapy. J Immunol Res.

[112] Karkada M, Berinstein NL, Mansour M (2014). Therapeutic vaccines and cancer: focus on DPX-0907. Biol Targets Ther.

[113] Bijker MS, van den Eeden SJ, Franken KL, Melief CJ, Offringa R, van der Burg SH (2007). CD8+ CTL priming by exact peptide epitopes in incomplete Freund's adjuvant induces a vanishing CTL response, whereas long peptides induce sustained CTL reactivity. J Immunol.

[114] Knutson KL, Schiffman K, Disis ML (2001). Immunization with a HER-2/neu helper peptide vaccine generates HER-2/neu CD8 T-cell immunity in cancer patients. J Clin Invest.

[115] Hos BJ, Tondini E, van Kasteren SI, Ossendorp F (2018). Approaches to improve chemically defined synthetic peptide vaccines. Front Immunol.

[116] Varypataki EM, Benne N, Bouwstra J, Jiskoot W, Ossendorp F (2017). Efficient eradication of established tumors in mice with cationic liposome-based synthetic long-peptide vaccines. Cancer Immunol Res.

[117] Kawakami Y, Eliyahu S, Sakaguchi K, Robbins PF, Rivoltini L, Yannelli JR (1994). Identification of the immunodominant peptides of the MART-1 human melanoma antigen recognized by the majority of HLA-A2-restricted tumor infiltrating lymphocytes. J Exp Med.

[118] Kotsakis A, Papadimitraki E, Vetsika EK, Aggouraki D, Dermitzaki EK, Hatzidaki D (2014). A phase II trial evaluating the clinical and immunologic response of HLA-A2+ non-small cell lung cancer patients vaccinated with an hTERT cryptic peptide. Lung Cancer.

[119] Sigalov AB (2014). A novel ligand-independent peptide inhibitor of TREM-1 suppresses tumor growth in human lung cancer xenografts and prolongs survival of mice with lipopolysaccharide-induced septic shock. Int Immunopharmacol.

[120] Ahsan A, Ramanand SG, Bergin IL, Zhao L, Whitehead CE, Rehemtulla A (2014). Efficacy of an EGFR-specific peptide against EGFR-dependent cancer cell lines and tumor xenografts. Neoplasia.

[121] Takahashi H, Okamoto M, Shimodaira S, Tsujitani S-i, Nagaya M, Ishidao T (2013). Impact of dendritic cell vaccines pulsed with Wilms' tumour-1 peptide antigen on the survival of patients with advanced non-small cell lung cancers. Eur J Cancer.

[122] Nishida S, Koido S, Takeda Y, Homma S, Komita H, Takahara A (2014). Wilms tumor gene (WT1) peptide-based cancer vaccine combined with gemcitabine for patients with advanced pancreatic cancer. J Immunother.

[123] Ishikawa N, Takano A, Yasui W, Inai K, Nishimura H, Ito H (2007). Cancer-testis antigen lymphocyte antigen 6 complex locus K is a serologic biomarker and a therapeutic target for lung and esophageal carcinomas. Cancer Res.

[124] Takahashi R, Toh U, Iwakuma N, Takenaka M, Otsuka H, Furukawa M (2014). Feasibility study of personalized peptide vaccination for metastatic recurrent triple-negative breast cancer patients. Breast Cancer Res.

[125] Ohtake J, Ohkuri T, Togashi Y, Kitamura H, Okuno K, Nishimura T (2014). Identification of novel helper epitope peptides of Survivin cancer-associated antigen applicable to developing helper/killer-hybrid epitope long peptide cancer vaccine. Immunol Lett.

[126] Parmiani G, Castelli C, Dalerba P, Mortarini R, Rivoltini L, Marincola FM (2002). Cancer immunotherapy with peptide-based vaccines: what have we achieved? Where are we going?. J Nat Cancer Inst.

[127] Smith TT, Moffett HF, Stephan SB, Opel CF, Dumigan AG, Jiang X (2017). Biopolymers codelivering engineered T cells and STING agonists can eliminate heterogeneous tumors. J Clin Invest.

[128] Chu Y, Liu Q, Wei J, Liu B (2018). Personalized cancer neoantigen vaccines come of age. Theranostics.

[129] Cheung AS, Zhang DKY, Koshy ST, Mooney DJ (2018). Scaffolds that mimic antigen-presenting cells enable *ex vivo* expansion of primary T cells. Nat Biotechnol.

[130] Singha S, Shao K, Ellestad KK, Yang Y, Santamaria P (2018). Nanoparticles for immune stimulation against infection, cancer, and autoimmunity. ACS Nano.

[131] Li H, Li Y, Wang X, Hou Y, Hong X, Gong T (2017). Rational design of Polymeric Hybrid Micelles to Overcome Lymphatic and Intracellular Delivery Barriers in Cancer Immunotherapy. Theranostics.

[132] Xu Y, Wang Y, Yang Q, Liu Z, Xiao Z, Le Z (2019). A versatile supramolecular nanoadjuvant that activates NF-kappaB for cancer immunotherapy. Theranostics.

[133] Wu Y, Norberg PK, Reap EA, Congdon KL, Fries CN, Kelly SH (2017). A supramolecular vaccine platform based on α-helical peptide nanofibers. ACS Biomater Sci Eng.

[134] Marincola FM, Jaffee EM, Hicklin DJ, Ferrone S (1999). Escape of human solid tumors from T-cell recognition: Molecular mechanisms and functional significance.

[135] Sasada T, Yamada A, Noguchi M, Itoh K (2014). Personalized peptide vaccine for treatment of advanced cancer. Curr Med Chem.

[136] Valmori D, Fonteneau J-F, Lizana CM, Gervois N, Liénard D, Rimoldi D (1998). Enhanced generation of specific tumor-reactive CTL *in vitro* by selected Melan-A/MART-1 immunodominant peptide analogues. J Immunol.

[137] Bezu L, Kepp O, Cerrato G, Pol J, Fucikova J, Spisek R (2018). Trial watch: Peptide-based vaccines in anticancer therapy. Oncoimmunology.

[138] Mohit E, Hashemi A, Allahyari M (2014). Breast cancer immunotherapy: monoclonal antibodies and peptide-based vaccines. Expert Rev Clin Immunol.

[139] Wang J, Hu X, Xiang D (2018). Nanoparticle drug delivery systems: an excellent carrier for tumor peptide vaccines. Drug Delivery.

[140] Silva AL, Rosalia RA, Sazak A, Carstens MG, Ossendorp F, Oostendorp J (2013). Optimization of encapsulation of a synthetic long peptide in PLGA nanoparticles: Low-burst release is crucial for efficient CD8+ T cell activation. Eur J Pharm Biopharm.

[141] Qiu F, Becker KW, Knight FC, Baljon JJ, Sevimli S, Shae D (2018). Poly (propylacrylic acid)-peptide nanoplexes as a platform for enhancing the immunogenicity of neoantigen cancer vaccines. Biomaterials.

[142] Hao J, Kwissa M, Pulendran B, Murthy N (2006). Peptide crosslinked micelles: a new strategy for the design and synthesis of peptide vaccines. Int J Nanomed.

[143] Zhang P, Chiu Y-C, Tostanoski LH, Jewell CM (2015). Polyelectrolyte multilayers assembled entirely from immune signals on gold nanoparticle templates promote antigen-specific T cell response. ACS Nano.

[144] Coolen A-L, Lacroix C, Mercier-Gouy P, Delaune E, Monge C, Exposito J-Y (2019). Poly (lactic acid) nanoparticles and cell-penetrating peptide potentiate mRNA-based vaccine expression in dendritic cells triggering their activation. Biomaterials.

[145] Zhong H, Chan G, Hu Y, Hu H, Ouyang D (2018). A comprehensive map of FDA-approved pharmaceutical products. Pharmaceutics.

[146] Tyler B, Gullotti D, Mangraviti A, Utsuki T, Brem H (2016). Polylactic acid (PLA) controlled delivery carriers for biomedical applications. Adv Drug Delivery Rev.

[147] Zhang Z, Tongchusak S, Mizukami Y, Kang YJ, Ioji T, Touma M (2011). Induction of anti-tumor cytotoxic T cell responses through PLGA-nanoparticle mediated antigen delivery. Biomaterials.

[148] Black M, Trent A, Kostenko Y, Lee JS, Olive C, Tirrell M (2012). Self-assembled peptide amphiphile micelles containing a cytotoxic T-Cell epitope promote a protective immune response *in vivo*. Adv Mater.

[149] Zhang L, Jing D, Wang L, Sun Y, Li JJ, Hill B (2018). Unique Photochemo-Immuno-Nanoplatform against Orthotopic Xenograft Oral Cancer and Metastatic Syngeneic Breast Cancer. Nano Lett.

[150] Muraoka D, Harada N, Hayashi T, Tahara Y, Momose F, Sawada S-i (2014). Nanogel-based immunologically stealth vaccine targets macrophages in the medulla of lymph node and induces potent antitumor immunity. ACS Nano.

